# Unique properties of dually innervated dendritic spines in pyramidal neurons of the somatosensory cortex uncovered by 3D correlative light and electron microscopy

**DOI:** 10.1371/journal.pbio.3001375

**Published:** 2021-08-24

**Authors:** Olivier Gemin, Pablo Serna, Joseph Zamith, Nora Assendorp, Matteo Fossati, Philippe Rostaing, Antoine Triller, Cécile Charrier

**Affiliations:** 1 Institut de Biologie de l’Ecole Normale Supérieure (IBENS), CNRS, INSERM, PSL Research University, Paris, France; 2 Laboratoire de Physique de l’Ecole Normale Supérieure, ENS, PSL Research University, CNRS, Sorbonne Université, Université Paris-Diderot, Sorbonne Paris Cité, Paris, France; Institute for Basic Science, REPUBLIC OF KOREA

## Abstract

Pyramidal neurons (PNs) are covered by thousands of dendritic spines receiving excitatory synaptic inputs. The ultrastructure of dendritic spines shapes signal compartmentalization, but ultrastructural diversity is rarely taken into account in computational models of synaptic integration. Here, we developed a 3D correlative light–electron microscopy (3D-CLEM) approach allowing the analysis of specific populations of synapses in genetically defined neuronal types in intact brain circuits. We used it to reconstruct segments of basal dendrites of layer 2/3 PNs of adult mouse somatosensory cortex and quantify spine ultrastructural diversity. We found that 10% of spines were dually innervated and 38% of inhibitory synapses localized to spines. Using our morphometric data to constrain a model of synaptic signal compartmentalization, we assessed the impact of spinous versus dendritic shaft inhibition. Our results indicate that spinous inhibition is locally more efficient than shaft inhibition and that it can decouple voltage and calcium signaling, potentially impacting synaptic plasticity.

## Abstract 2

Pyramidal neurons are covered by thousands of dendritic spines receiving excitatory synaptic inputs. A new 3D correlative light-electron microscopy (3D-CLEM) approach enables the cartography of synaptic ultrastructure with cell type-, dendrite type- and synapse type-specific resolution in brain tissues, revealing unique properties of spines that receive dual innervation.

## Introduction

In the mammalian cortex, the vast majority of excitatory synapses are formed on dendritic spines, small membrane protrusions that decorate the dendrites of pyramidal neurons (PNs) [1–3]. Dendritic spines are composed of a bulbous head connected to the dendritic shaft by a narrow neck [4,5]. They exist in a large variety of shapes and sizes along individual dendrites. Spine head volume can vary between 3 orders of magnitude (0.01 to 1.5 μm^3^), neck length between 0.2 μm and 3 μm, and minimal neck diameter between 20 and 500 nm [6]. Spine heads are typically contacted by an excitatory synaptic input and harbor an excitatory postsynaptic density (ePSD) that contains glutamatergic α-amino-3-hydroxy-5-methyl-4-isoxazolepropionic acid (AMPA) and N-Methyl-D-aspartate (NMDA) neurotransmitter receptors, scaffolding proteins, adhesion molecules, and a complex machinery of proteins undertaking the transduction of synaptic signals. The size of the spine head correlates with the size of the ePSD and the strength of synaptic transmission [7–11]. In addition to the ePSD, spines contain ribosomes, which mediate local protein synthesis, and endosomes, which play a critical role in membrane and receptor trafficking [12,13]. The largest spines often contain a spine apparatus (SA), which contributes to calcium signaling and synaptic plasticity [12,14], and some spines, especially in the upper layers of the cortex, also house an inhibitory postsynaptic specialization [15]. Spine necks are diffusional barriers that biochemically isolate spine heads from their parent dendrite [16–19]. In addition, they can filter the electrical component of synaptic signals and amplify spine head depolarization [20–22] (but see [23–25]). Both spine heads and spine necks are remodeled depending on neuronal activity [9,26,27] and in pathology [28,29]. While the relationship between spine morphology and function is widely acknowledged, and although dendritic spines are known to participate in different neural circuits depending on their location in the dendritic tree [30], the extent of synaptic ultrastructural diversity along individual identified dendrites has not been quantified, and the consequences of this variability on signal compartmentalization and dendritic integration remain to be investigated.

Dendritic signaling can be modeled based on anatomical and biophysical parameters [31] using “realistic” multicompartment models [32]. These models were pioneered by Wilfrid Rall following the seminal works of Hodgkin and Huxley [33,34]. They have provided a powerful theoretical framework for understanding dendritic integration [35], spine function [36], inhibitory signaling [37,38], and electrical compartmentalization in spines [22,39,40]. However, spines and synapses are usually modeled with *ad hoc* or averaged biophysical parameters, which limit the accuracy of the prediction [41]. Modeling the actual behavior of dendritic spines requires an accurate description of their ultrastructural heterogeneity with a cell-type- and dendritic-type-resolution. To acquire such data, it is necessary to combine the nanometer resolution of electron microscopy (EM) with an approach that allows the identification of the origin of dendritic spines (i.e., location on the dendrite, type of dendrite, and type of neuron) without obscuring the intracellular content. This task is arduous: 1 mm^3^ of mouse cortex contains over 50,000 of neurons, each of which establishes approximately 8,000 synaptic connections with neighboring neurons, and these synapses are highly specific, connecting multiple neuronal subtypes from various brain regions [42–45]. Reconstructing selected dendritic spines and synaptic contacts along dendritic trees requires either enormous volumes of 3D-EM acquisitions using resource-consuming approaches adapted from connectomics [46–49] or combining EM with a lower-scale imaging modality such as confocal or 2-photon light microscopy (LM) to guide 3D-EM image acquisitions to the region of interest (ROI) [50–52]. While very powerful in vitro [50,53,54], correlative light–electron microscopy (CLEM) is difficult to implement in brain tissues [55–57]. New protocols are required to facilitate the *in situ* identification of targeted dendrites and synapses in different imaging modalities and to make 3D-CLEM more accessible to the neuroscientific community.

Here, we have developed a CLEM workflow combining confocal light microscopy with serial block–face scanning EM (SBEM) and targeted photoprecipitation of 3,3-diaminobenzidine (DAB) to facilitate ROI recovery. We applied this workflow to reconstruct dendritic spines located exclusively on the basal dendrites of genetically labelled PNs in layer 2/3 (L2/3) of the somatosensory cortex (SSC) of adult mice. We analyzed the variability of their ultrastructure and estimated the electrical resistance of their neck. We also examined the distribution and the morphology of inhibitory synapses. We specifically examined dendritic spines receiving both excitatory and inhibitory inputs, which represented 10% of all spines along basal dendrites. These dually innervated spines (DiSs) exhibited wider heads and larger ePSDs than singly innervated spines (SiSs), and they were more electrically isolated from the dendritic shaft than SiSs of comparable head size. We then used our measurements to constrain a multicompartment model of synaptic signaling and compartmentalization in dendrites. We assessed the effects of individual excitatory and inhibitory signals on membrane voltage and calcium concentration depending on inhibitory synapse placement (i.e., on a spine head or on the dendritic shaft) and input timing. Our results challenge the view that spinous inhibition strictly vetoes single excitatory inputs and rather suggest that it fine-tunes calcium levels in DiSs. Our simulations indicate that a single inhibitory postsynaptic potential (IPSP) evoked in a DiS within 10 ms after an excitatory postsynaptic potential (EPSP) can curtail the local increase of calcium concentration without affecting the amplitude of membrane depolarization. This decoupling effect could impact long-term synaptic plasticity in cortical circuits.

## Results

### Combining light and electron microscopy to access the ultrastructure of targeted populations of dendritic spines in brain slices

**Fig 1:**
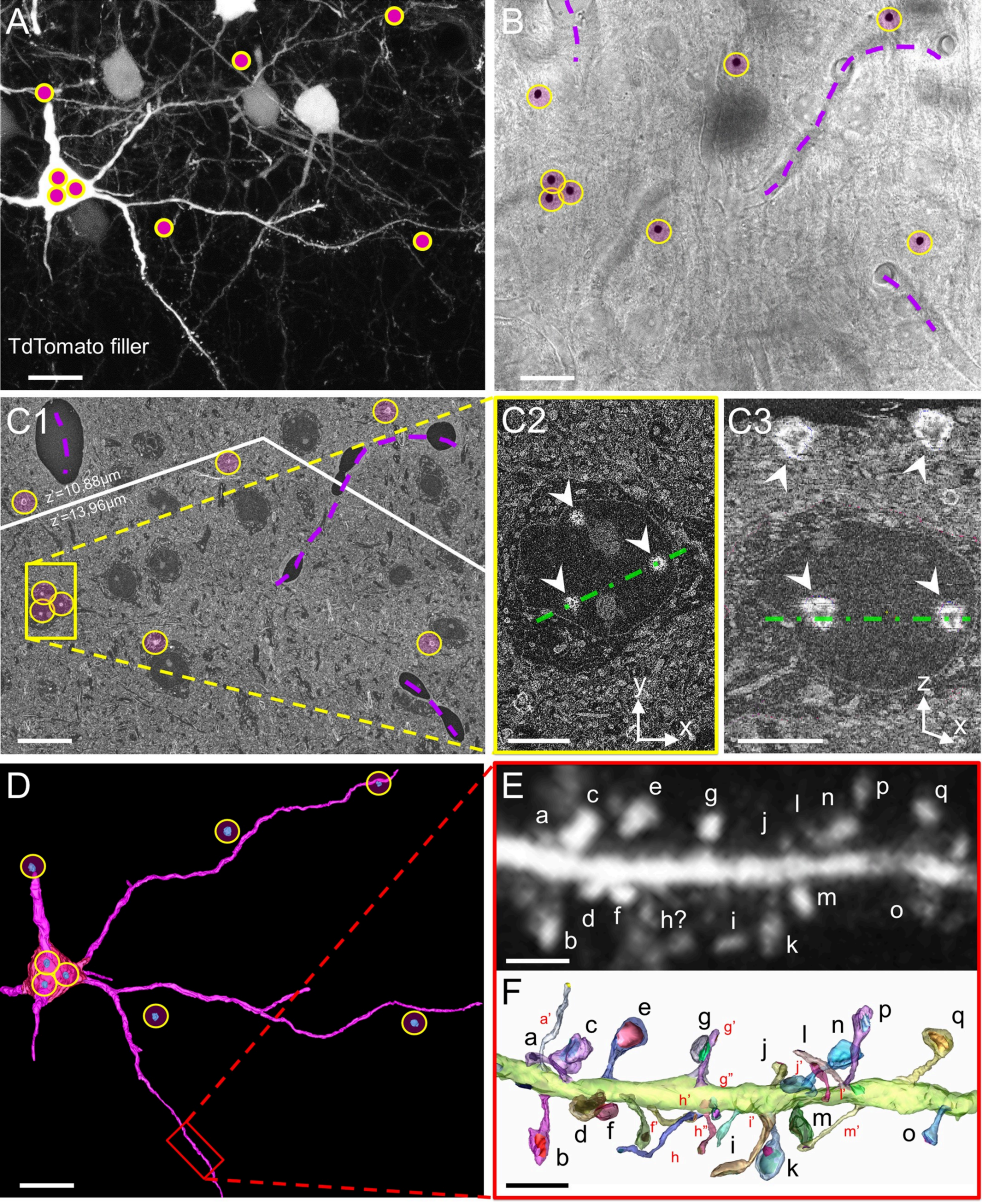
CLEM imaging of identified spines within intact cortical circuits. (A) Visualization of basal dendrites of a PN expressing cytosolic TdTomato in L2/3 of adult mouse SSC. DAB was photoprecipitated using focused UV light to insert correlative landmarks (pink dots in yellow circles). (B) Transmitted light image of the same field of view after DAB photoprecipitation. DAB precipitates are highlighted with yellow circles. Blood vessels are outlined with purple dashed lines. (C) Composite SEM image displaying DAB patterning at the depth of the neuron of interest (yellow circles). Slight mismatch between LM and SEM observation planes resulted in DAB landmarks appearing in different z-planes during block facing; the white line represents stitching between z-shifted images. In C1, landmarks are arranged as in B. C2 is a close-up on the soma of the electroporated neuron, labelled with 3 DAB landmarks (arrowheads). C3 is an orthogonal (x,z) view of the SEM stack along the axis represented as a green dashed line in C2 and C3. The superficial DAB layer enabled ROI targeting, and the deeper layer enabled retrospective identification of the target neuron. (D) 3D reconstruction of dendrites of interest from the overview SEM stack. DAB landmarks are reconstructed in blue (in yellow circles). The red rectangle outlines the portion of dendrite represented in E and F. (E) Z-projection of the confocal stack corresponding to the portion of dendrite reconstructed in D. Letters identify individual spines. (F) 3D-EM reconstruction. Individual dendritic spines were manually segmented and randomly colored. Spines that were detected in CLEM but not in LM alone are labelled in red. Scale bars: A, B, C1, D: 10 μm; C2, C3: 5 μm; E, F: 2 μm. CLEM, correlative light–electron microscopy; DAB, 3,3-diaminobenzidine; LM, light microscopy; L2/3, layer 2/3; PN, pyramidal neuron; ROI, region of interest; SEM, scanning EM; SSC, somatosensory cortex; 3D-EM, three-dimensional electron microscopy.

In the cortex, the morphology and distribution of dendritic spines vary depending on cortical area and layer in which the cell body is located [5,35,58,59], and dendritic spines are differently regulated depending on their location within dendritic trees—e.g., basal or apical dendrites [30,49,60–62]. Therefore, it is critical to take into account both the cellular and dendritic context to characterize the diversity of spine ultrastructure. To that aim, we developed a 3D-CLEM workflow allowing the ultrastructural characterization of dendritic spines on genetically defined neuronal cell types and along identified types of dendrites in intact cortical circuits. In order to sparsely label specific subtypes of neurons, we used cortex-directed *in utero* electroporation (IUE) in mice. We electroporated neuronal progenitors generating L2/3 cortical PNs at embryonic day (E)15.5 with a plasmid expressing the fluorescent cytosolic filler tdTomato, granting access to the morphology of electroporated neurons, their dendrites, and their dendritic spines in LM. We perfused adult mice with aldehyde fixatives and collected vibratome sections of the electroporated area. To facilitate sample handling, we designed custom-made chambers allowing sample immersion in different solutions during confocal imaging and subsequent retrieval of the sample before EM preparation steps (S1 Fig). We enclosed 10 to 20 mm^2^ fragments of brain sections in these chambers and acquired images of optically isolated basal dendrites of bright electroporated neurons with confocal microscopy (Fig 1A).

A major challenge of CLEM in brain tissue is to recover the ROI in EM after imaging in LM. Several methods have been proposed to facilitate ROI recovery [50–52], but they come with the following caveats: (1) using only intrinsic landmarks has a low throughput [57,63]; (2) filling target neurons with DAB masks intracellular ultrastructure [64]; and (3) scarring the tissue with an infrared laser to generate extrinsic landmarks, a.k.a. “NIRB” for “near-infrared branding” [56,65–68], produces landmarks with low pixel intensity in EM and can damage ultrastructure [63,69]. To facilitate ultrastructural measurements in non-obscured identified dendrites, we took advantage of the photo-oxidability of DAB [70,71]. We immersed the samples in DAB solution and applied focalized UV light at user-defined positions (Fig 1A) to imprint osmiophilic DAB landmarks around targeted dendrites (see S1B–S1E Fig) and pattern the tissue with localized electron-dense DAB precipitates (Fig 1B). After sample retrieval (see panel F in S1 Fig), tissue sections were processed for SBEM and embedded in minimal amounts of epoxy resin in order to maximize sample conductivity and SBEM image quality (see Materials and methods). In 3D-EM stacks, ROIs were recovered within the complex environment of brain tissues using both intrinsic landmarks such as blood vessels (Fig 1B) and high-contrast DAB precipitates (Fig 1C; see also panel G in S1 Fig). We then segmented and reconstructed targeted dendrites in 3D (Fig 1D) and registered whole portions of dendrites in both LM and EM to identify each dendritic spine unequivocally using neighboring spines as dependable topographic landmarks (Fig 1E and 1F). CLEM-based 3D reconstruction enabled the identification of dendritic spines that were not visible in LM or EM alone. In LM, the limited axial resolution prevents the identification of axially oriented spines, which are easily detected in 3D-EM [49] (Fig 1E and 1F; see also corresponding movie at https://www.opendata.bio.ens.psl.eu/3DCLEM-Spines/S1_Movie.zip; login: guest, password: EnsData0811). On the other hand, laterally oriented spines with the longest and thinnest necks are conspicuous in LM stacks but can be difficult to find in 3D-EM datasets without the cues provided by LM. The proportion of spines recovered with CLEM versus LM alone could amount to up to 30% per ROI, and 5% per ROI versus EM alone, highlighting the advantage of CLEM over unimodal microscopy approaches.

### Spine ultrastructure along the basal dendrites of L2/3 cortical pyramidal neurons

**Fig 2:**
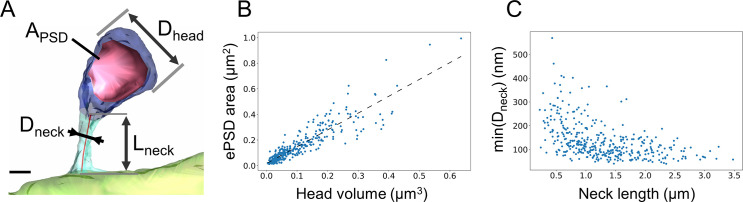
Spine morphometry along basal dendrites of L2/3 cortical PNs. (A) 3D reconstruction of a dendritic spine from an SBEM stack. Dendritic shaft is in light green, spine neck in turquoise, spine head in blue, and PSD surface in red. The following parameters were measured: PSD area, head diameter, neck diameter, and neck length. Scale bar: 300 nm. (B) Linear correlation of PSD area and spine head volume. R^2^ = 0.82. (C) Plot of the minimal spine neck diameter as a function of spine neck length. Spearman correlation coefficient is −0.58. *N =* 390. The data underlying this figure can be found in https://www.opendata.bio.ens.psl.eu/3DCLEM-Spines/data/Data_related_to_Fig 2.xlsx (login: guest; password: EnsData0811). ePSD, excitatory postsynaptic density; L2/3, layer 2/3; PN, pyramidal neuron; PSD, postsynaptic density; SBEM, serial block–face scanning EM.

We used our CLEM workflow to quantify the full extent of the ultrastructural diversity of dendritic spines along the basal dendrites of L2/3 PNs of the SSC of 3 adult mice. We exhaustively segmented 254 μm of the basal dendritic arborization of 4 neurons, and we reconstructed a total of 390 individual spines (S1 Data). As spine distance to the soma spanned from 20 to 140 μm, with basal dendrites extending up to 150 μm [72–75], our dataset can be considered representative of the whole spine population on these dendrites. The average linear density of dendritic spines was 1.5 ± 0.3 spine.μm^−1^. We then quantified the following parameters for each spine: neck length, neck diameter, head volume, head longitudinal diameter (referred to as “head length”), head orthogonal diameter (referred to as “head diameter”), number of PSDs, and PSD area (Fig 2A, S1 Data). In agreement with previous reports in both basal and apical dendrites of mouse cortical and hippocampal neurons [4,6,76,77], we found that ePSD area correlates linearly with the volume of the spine head (Fig 2B). We also observed a nonlinear correlation between the length of the spine neck and its diameter (Fig 2C): Long spines (neck length > 2 μm) always had thin necks (neck diameter < 0.2 μm), although short necks could also be thin. By contrast, there was no correlation between the position of the spine or the interspine distance and any of the morphological parameters we measured (S2 Fig). There was also no correlation between the length or the diameter of the neck and the morphometry of the spine head or ePSD (S1 Data), which is consistent with previous EM studies of L2/3 PNs of mouse neocortex [4,72] (but see [40,78] for different conclusions in other brain areas).

**Fig 3:**
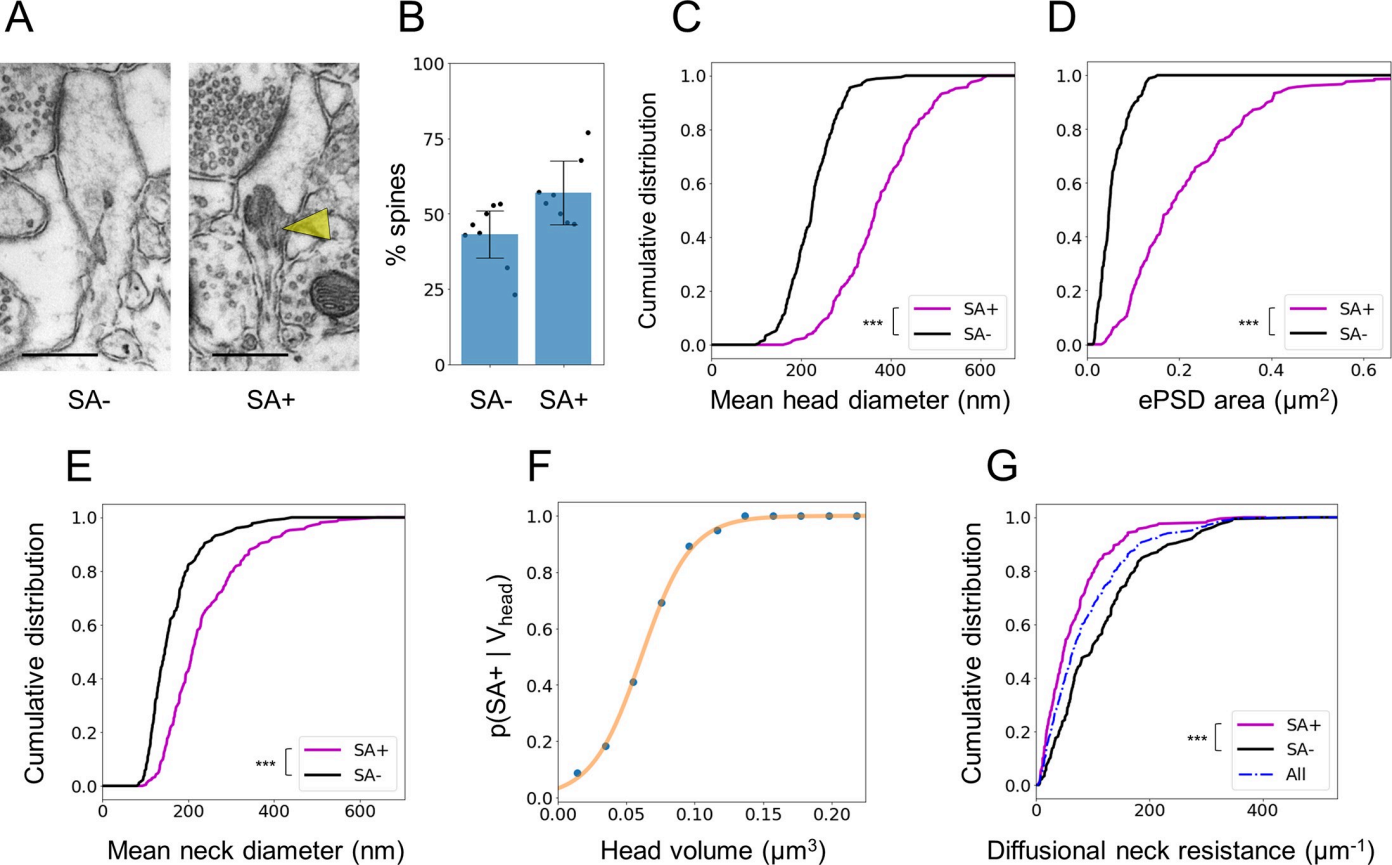
Spines containing an SA have larger head and wider neck. (A) TEM images of spines either devoid of SA (left) or containing an SA (SA+) (right, yellow arrowhead). Scale bars: 500 nm. (B) Proportion of SA− and SA+ spines. Histogram represents mean ± SD, from 390 spines in *N =* 8 dendrites. (C) Distribution of mean head diameter for SA− and SA+ spines. *N* = 179 and 221, respectively (*p* < 10^−38^). (D) Distribution of ePSD area. (*p* < 10^-40^). (E) Distribution of mean neck diameter. (*p* < 10^-12^). (F) Probability of harboring an SA as a function of spine head volume. Blue: experimental data. Orange: sigmoid fit. (G) Distribution of the diffusional resistance of the spine neck (W_neck_) calculated based on neck morphology (*p* < 10^−5^). ***: *p* < 0.001 calculated using Mann–Whitney test. The data underlying this figure can be found in https://www.opendata.bio.ens.psl.eu/3DCLEM-Spines/data/Data_related_to_Fig 3.xlsx (login: guest; password: EnsData0811). ePSD, excitatory postsynaptic density; SA, spine apparatus; TEM, transmission electron microscopy.

Since our CLEM approach grants access to the cytosolic content of spines (Fig 3A), we quantified the occurrence of SA, a complex stacked membrane specialization of smooth endoplasmic reticulum (SER), which contributes to calcium signaling, integral membrane protein trafficking, local protein synthesis, and synaptic plasticity [12–14,79,80]. In basal dendrites, about 54% of spines contained an SA (Fig 3B), which is substantially higher than previous reports in the mature hippocampus [12,81]. These spines were randomly distributed along the dendrites. They had larger heads (Fig 3C), larger ePSDs (Fig 3D), and wider necks than spines devoid of SA (Fig 3E), consistent with previous morphological studies of CA1 PNs [12,81,82]. The probability that a spine contained an SA depending on spine head volume followed a sigmoid model (Fig 3F), predicting that all spines with a head diameter larger than 1.1 μm (21% spines in our reconstructions) contain an SA.

Next, we used our ultrastructural data to estimate the electrical resistance of spine necks using R_neck_ = ρ W_neck_, where ρ is the cytosolic resistivity (set to 300 Ω.cm [83,84]) and W_neck_ is the diffusional neck resistance that restricts the diffusion of molecules and charges between spine heads and dendritic shafts [23]. To quantify W_neck_, for each spine, we measured a series of orthogonal cross-sections of the neck along its principal axis and integrated W_neck_ = ∫ dℓ / A(ℓ), where A(ℓ) is the neck cross-section area at the abscissa ℓ along the neck axis. W_neck_ ranged from 2 μm^−1^ to 480 μm^−1^ and R_neck_ from 8 MΩ to 1450 MΩ, with a median value of 188 MΩ. These values are consistent with previous estimations based on EM reconstructions and stimulated emission depletion (STED) super-resolutive light microscopy [17,85] and with direct electrophysiological recordings [86]. It has been proposed that the SA, which may occupy some of the spine neck volume, could increase W_neck_ [13,79,87]. Therefore, we subtracted SA cross-section from A(ℓ) when computing W_neck_ in SA+ spines (see Materials and methods). This correction increased W_neck_ by 13% ± 2% in SA+ spines (S3 Fig). However, because of their wider necks, W_neck_ of SA+ spines was still lower (59% in average) than W_neck_ of spines devoid of SA (Fig 3G). These results suggest that, in addition to supplying large dendritic spines with essential resources, the SA may adjust W_neck_ and influence spine compartmentalization [12,13,81].

### Excitatory and inhibitory synapses in dually innervated spines

**Fig 4:**
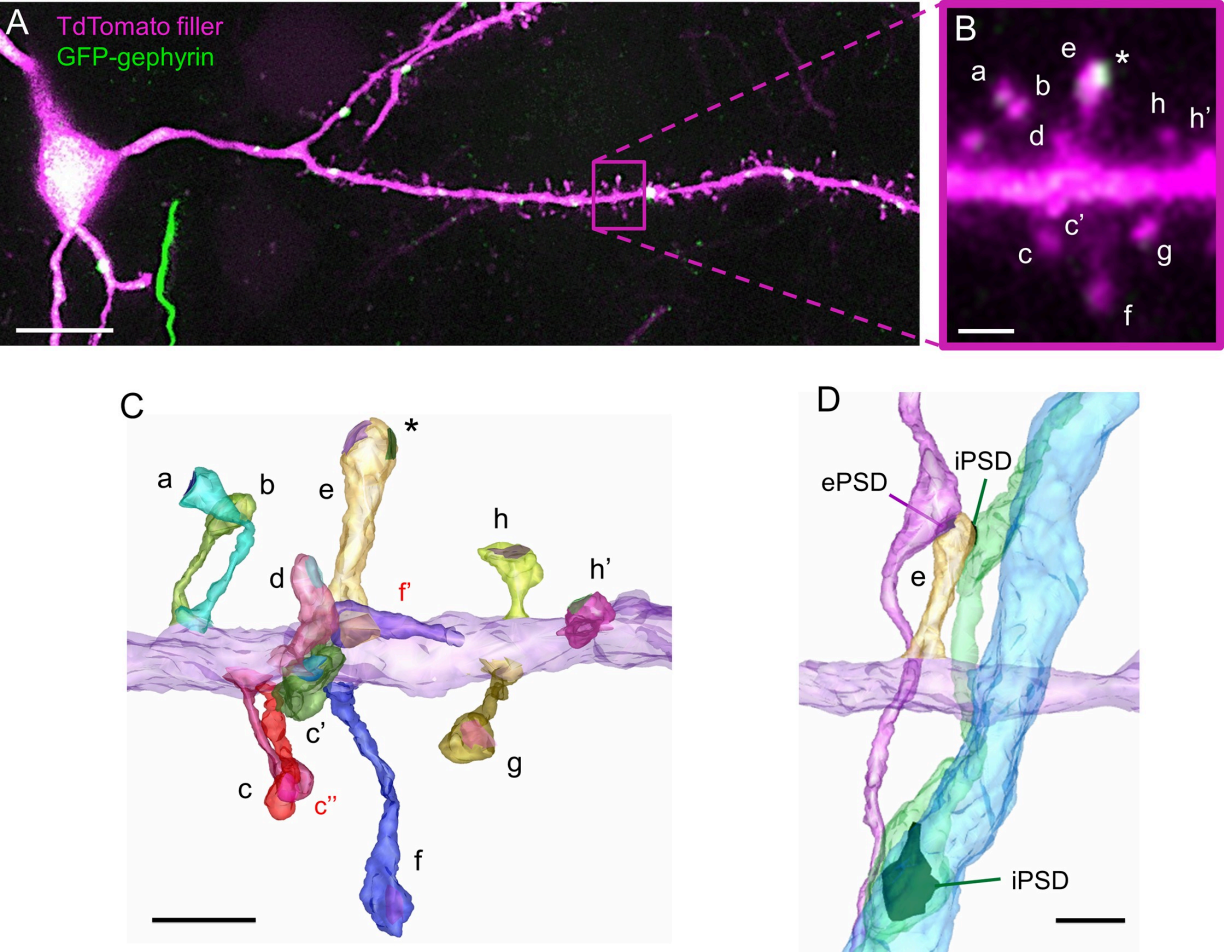
Identification of excitatory and inhibitory synapse on DiSs using CLEM. (A) Confocal image of basal dendrites of a cortical L2/3 PN that was electroporated with cytosolic TdTomato and GFP-GPHN to label inhibitory synapses. The magenta rectangle outlines the region enlarged in B. (B) Enlargement of a portion of the dendrite in A harboring several dendritic spine (lettered). Spine “e” contains a cluster of GFP-GPHN (asterisk) and corresponds to a putative DiS. (C) 3D-EM reconstruction of the same dendritic fragment as in B. Dendritic shaft is colored in purple; individual spines and PSDs are colored randomly. Spines visible in EM but not in LM are labelled in red. The iPSD (colored in green) on spine “e” is identified based on the position of the GFP-GPHN cluster (asterisk in B and C). GFP-GPHN-negative PSDs are defined as excitatory. (D) 3D-EM reconstruction of spine “e” (yellow) with its presynaptic partners (magenta and green). As the “green” axon also targets a neighboring dendritic shaft (blue), it is defined as inhibitory. Scale bars: A: 10 μm; B, C, D: 1 μm. CLEM, correlative light–electron microscopy; DiS, dually innervated spine; EM, electron microscopy; ePSD, excitatory postsynaptic density; GFP-GPHN, GFP-tagged gephyrin; iPSD, inhibitory PSD; LM, light microscopy; L2/3, layer 2/3; PN, pyramidal neuron; PSD, postsynaptic density; 3D-EM, three-dimensional electron microscopy.

We noticed that a small proportion of dendritic spines were contacted by 2 distinct presynaptic boutons (DiSs). DiSs have long been described in the literature as receiving both an excitatory and an inhibitory synaptic contact [88–91]. In the SSC, DiSs are contacted by VGLUT2-positive thalamocortical inputs [15], and they are sensitive to sensory experiences. The number of DiSs increases in response to sensory stimulation and decreases in response to sensory deprivation [73,92–94], suggesting their importance in synaptic integration and sensory processing. However, their scarcity in the cortex has been an obstacle to their ultrastructural and functional characterization. We took advantage of our CLEM approach and the molecular signature of this population of spines (i.e., the presence of a cluster of gephyrin, the core protein of inhibitory postsynaptic scaffolds [95,96]) to examine their morphological properties. To label inhibitory synapses in cortical PNs (Fig 4A), we co-expressed tdTomato with small amounts of GFP-tagged gephyrin (GFP-GPHN) [73,94,97,98]. We identified in LM spines containing a gephyrin cluster (Fig 4B), and we ascertained their dual innervation in EM after back-correlating spine identity between LM and SBEM acquisitions. To do so, we aligned reconstructed dendrites on LM images (Fig 4C) and matched individual spines in both modalities (lettered in Fig 4B and 4C). While ePSDs look asymmetrical and more electron dense than inhibitory PSDs (iPSDs) in transmission EM [99,100], the anisotropic resolution of SBEM does not allow the distinction of ePSDs and iPSDs in most DiSs [49]. Therefore, we identified iPSDs on DiSs based on GFP-GPHN cluster position in LM images. In 89% of DiSs (33/37), the excitatory (GFP-GPHN-negative) PSD and the inhibitory (GFP-GPHN-positive) PSD could be clearly discriminated. However, in 11% of DiSs (4/37 DiSs), distinguishing ePSD from iPSD was not obvious due to the coarse axial resolution of LM imaging. To resolve ambiguities, we reconstructed the axons innervating the DiSs and determined their identity based on their other targets in the neuropil, either soma and dendritic shaft for inhibitory axons [2,101,102], or other dendritic spines for excitatory axons [49] (Fig 4D). As a result, we could unequivocally determine the excitatory or inhibitory nature of each synaptic contact on electroporated neurons, within approximately 10^5^ μm^3^ 3D-EM acquisition volume.

**Fig 5:**
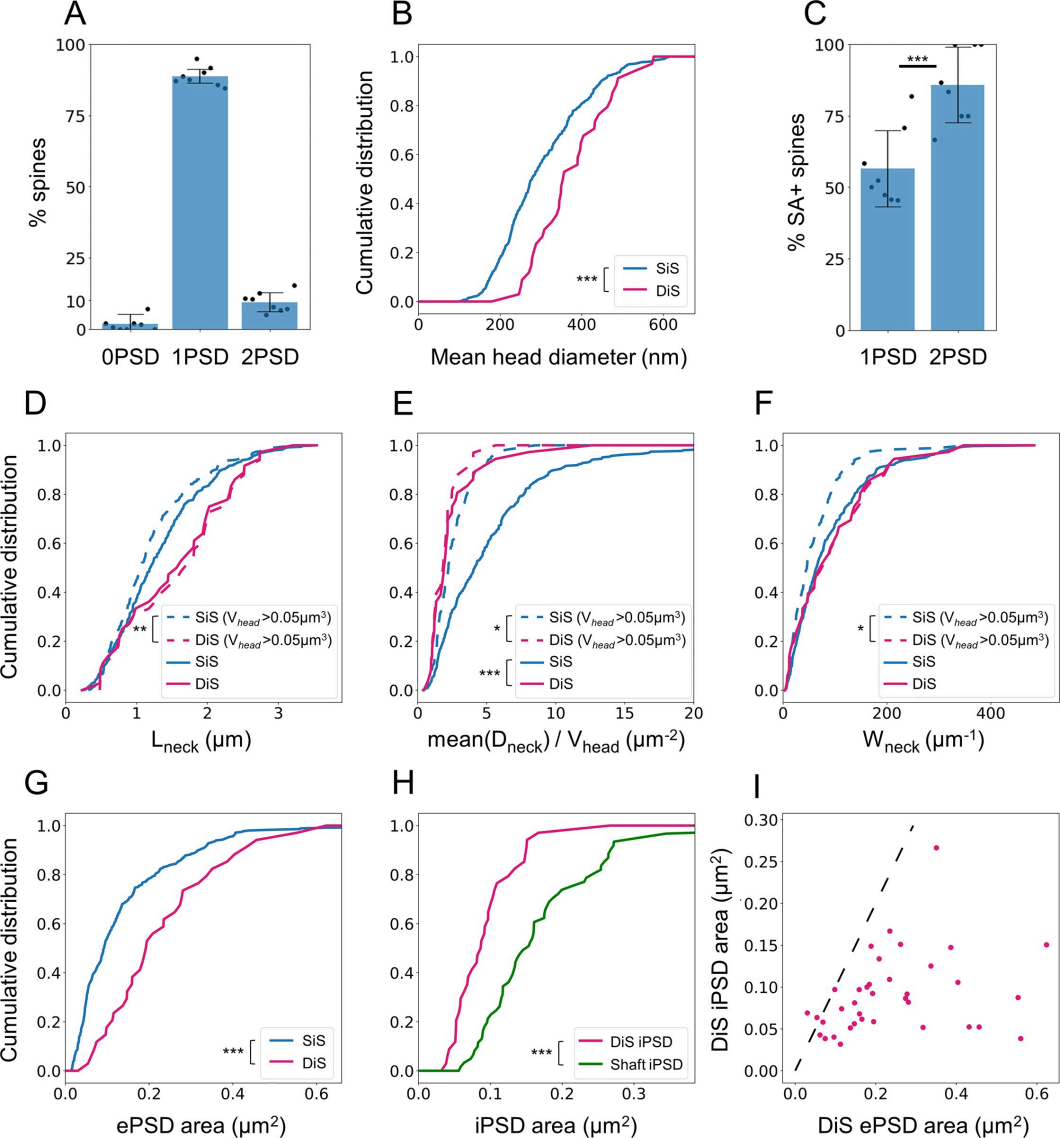
DiSs have unique anatomical properties. (A) Proportion of spines harboring 0, 1, or 2 synaptic contacts, quantified with CLEM. Histograms represent mean ± SD, from 390 spines in *N =* 8 dendrites. (B) Quantification of mean spine head diameter for SiSs (blue) and DiSs (red) (*p* < 10^−4^). (C) Proportion of SiSs and DiSs harboring an SA (*p* < 10^−10^ using Pearson χ^2^ test). (D-F) Quantification of neck length (D), the ratio between mean neck diameter and head volume (E), and the diffusional neck resistance (W_neck_) (F) between SiSs and DiSs (solid lines, *N* = 349 and 37, respectively) and between DiSs with SiSs of similar head volume (spines with V_head_ > 0.05 μm^3^, dashed lines, *N* = 186 and 34, respectively). (G) Quantification of ePSD area in SiSs or DiSs (*p* < 10^−5^). (H) Quantification of iPSD area in DiSs and dendritic shafts. *N* = 37 and 62, respectively (*p* < 10^−6^). (I) Plot of iPSD area as a function of ePSD area in individual DiSs. The dashed line (y = x) highlights that the ePSD is larger than the iPSD in most of DiSs. *N* = 37. *p*-values were computed using Mann–Whitney test (B, D-H) or Pearson χ^2^ test (C). Only significant (*p* < 0.05) *p*-values are shown (*p < 0.05; **p < 0.01; ***p < 0.001). The data underlying this figure can be found in https://www.opendata.bio.ens.psl.eu/3DCLEM-Spines/data/Data_related_to_Fig 5.xlsx (login: guest; password: EnsData0811). CLEM, correlative light–electron microscopy; DiSs, dually innervated spines; ePSD, excitatory postsynaptic density; iPSD, inhibitory PSD; PSD, postsynaptic density; SA, spine apparatus; SiSs, singly innervated spines.

In CLEM, we measured an average density of 1.4 ± 0.5 iPSDs per 10 μm of dendrite on DiSs and 2.1 ± 1.2 iPSDs per 10 μm of dendrite on the dendritic shaft—amounting to 3.5 ± 1.1 iPSDs per 10 μm of dendrite. iPSDs were homogeneously distributed either on spines or shaft from 24 μm away from the soma to the dendritic tip, which contrasts with apical dendrites where spinous inhibitory synapses are distally enriched [73]. Along the basal dendrites of L2/3 cortical PNs, 38% of inhibitory contacts occurred on dendritic spines, which is higher than previously estimated [73,98]. This percentage was stable from P21 but lower in young (P10) neurons and in layer 5 PNs (S4 Fig). DiSs represented 10% ± 3% of all spines (Fig 5A). They had larger heads than SiSs (Fig 5B), in line with previous reports [15,103], and 86% ± 13% of them contained an SA (Fig 5C). DiSs also differed in terms of neck morphology. They had longer necks than SiSs of comparable head volume (V_head_ > 0.05 μm^3^), although neck length distribution was similar in the whole populations of SiSs and DiSs (Fig 5D). DiSs also had lower D_neck_/V_head_ ratio than SiSs (Fig 5E), although D_neck_ distribution was similar between SiSs and DiSs (S5 Fig), suggesting that excitatory signals generated in DiSs are more compartmentalized than signals of similar amplitude generated in SiSs. Accordingly, DiSs had a higher W_neck_ than SiSs of comparable head size (52% larger in average) (Fig 5F). In spine heads, ePSDs on DiSs were larger than ePSDs on SiSs (174% ± 113% of ePSD area) (Fig 5G), consistent with the larger head size of DiSs. By contrast, iPSDs on DiSs were smaller than shaft iPSDs (53% ± 15% of shaft iPSD area) (Fig 5H). The area of iPSDs on DiSs did not correlate with spine head volume (S6 Fig). In 95% of DiSs, iPSDs were smaller than ePSDs (half the area, in average) (Fig 5I). Together, these results indicate that DiSs represent a specific population of dendritic spines with distinctive ultrastructural features that could impact their functional properties.

### Morphologically constrained modeling of synaptic signaling

**Fig 6:**
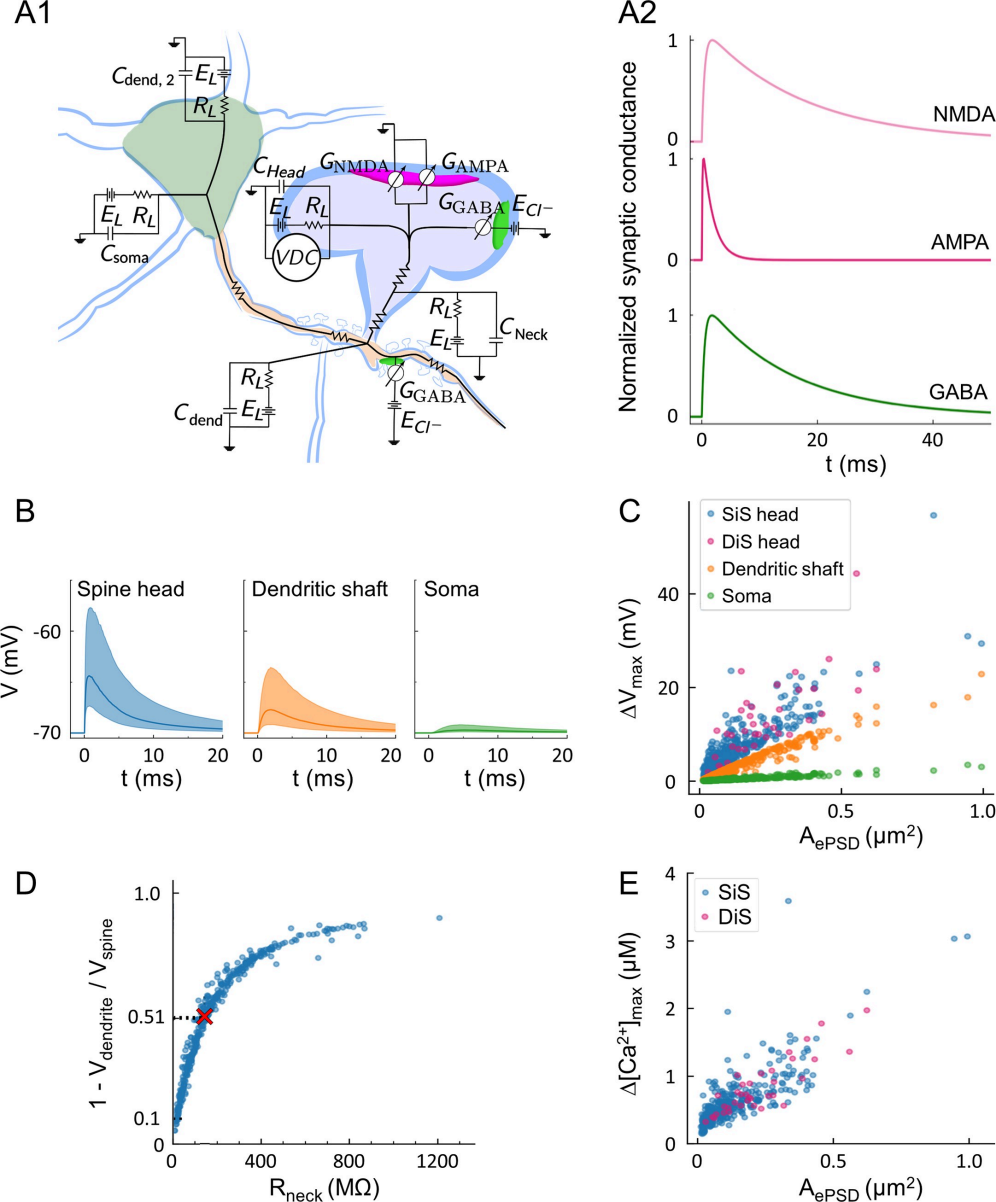
Morphologically constrained modeling of synaptic signaling. (A) Schematic of the circuit model (A1) and representative time course of excitatory (magenta) and inhibitory (green) conductance based on the kinetics of AMPA, NMDA, and GABA_A_ receptors (A2). All compartments include passive RC circuits to model cell membrane properties and optionally include an active conductance that models VDC. All modeled spines feature an excitatory synapse with glutamatergic AMPA and NMDA currents. Spines and dendritic compartments can also feature an inhibitory synapse with GABAergic currents. All conductances were scaled to PSD area (see Materials and methods). (B) Simulation of the time courses of membrane depolarization following an EPSP, taking into account spine diversity (i.e., R_neck_, ePSD area, and distance to soma, as measured in CLEM) in the spine head (blue), in the dendritic shaft in front of the spine (orange), and in the soma (green). (C) Amplitude of evoked depolarization (ΔV_max_) as a function of ePSD area at 3 distinct locations: head of SiSs (blue) or DiSs (magenta) where the EPSP was elicited, dendritic shaft 1 μm from the spine (orange) or soma (green). (D) Attenuation of the amplitude of depolarization between the spine head and the dendrite as a function of the resistance of the neck (R_neck_). The attenuation was calculated as: α = 1 − ΔV_max, shaft_ / ΔV_max, spine_. Red cross: mean value of α. (E) Estimated amplitude of intracellular calcium concentration transients Δ[Ca^2+^]_max_, following activation of NMDA receptors and VDCCs as a function of ePSD area. Three spiking outliers are not represented. The data underlying this figure can be found in https://www.opendata.bio.ens.psl.eu/3DCLEM-Spines/data/Data_related_to_Fig 6.xlsx (login: guest; password: EnsData0811). AMPA, α-amino-3-hydroxy-5-methyl-4-isoxazolepropionic acid; CLEM, correlative light–electron microscopy; DiSs, dually innervated spines; ePSD, excitatory postsynaptic density; EPSP, excitatory postsynaptic potential; NMDA, N-Methyl-D-aspartate; PSD, postsynaptic density; RC, resistor–capacitor; SiSs, singly innervated spines; VDC, voltage-dependent current; VDCC, voltage-dependent calcium channel.

Next, we wanted to assess the impact of spine diversity on synaptic signals. We used a computational approach based on a multicompartment “ball-and-stick” model of the neuronal membrane [40,104]. This model comprises an isopotential soma and 2 dendritic compartments structured as cables featuring passive resistor–capacitor (RC) circuits and conductance-based synapses. The 2 dendritic compartments correspond to the dendrite receiving the synaptic inputs and to the remainder of the dendritic tree (Fig 6A1) [105,106]. We constrained this model with morphological parameters measured in CLEM (i.e., for 390 spines and 37 DiSs: spine head volume and membrane area, ePSD and iPSD area, neck resistance, distance between spine and soma, dendritic diameter), taking into account the structural shrinkage resulting from chemical fixation (see S7 Fig). Individual synaptic AMPA, NMDA, and GABA_A_ conductances (Fig 6A2) were scaled proportionally to PSD areas [77,107–110]. Voltage-dependent calcium channels (VDCCs) were modeled in spine heads using Goldman–Hodgkin–Katz equations [111], and their conductance was scaled proportionally to spine head areas. Conductances were adjusted to fit published electrophysiological values (see Materials and methods) [37,38,112–122].

We first examined the propagation of simulated EPSPs. The amplitude of the depolarization evoked in spine heads followed a log-normal distribution reflecting the morphological variability of spines (Fig 6B). The maximal amplitude of the depolarization (ΔV_max_) was sharply attenuated between the head of the spine and the dendritic shaft (51% attenuation in average), and about 5% of ΔV_max_ reached the soma (Fig 6B and 6C), which is in the range of measurements performed in basal dendrites of L5 cortical PNs using voltage dyes, electrophysiology, and glutamate uncaging [25,123]. To determine the contribution of individual morphological parameters to the variance of ΔV_max_ in spine heads, we used a generalized linear model (GLM) [124]. In SiSs, A_ePSD_ and R_neck_ accounted for 60% and 19% of the variance of ΔV_max_, respectively (also see S8 Fig for the dependence of ΔV_max_ on R_neck_). In DiSs, the contribution of R_neck_ to ΔV_max_ was much higher, reaching 38% of the variance, while A_ePSD_ contribution dropped to 47% (S1 Table). In 56% of dendritic spines, R_neck_ was large enough (>145 MΩ) to attenuate EPSP amplitude by >50% across the spine neck, and more than 90% of spine necks attenuated the signal by at least 10% (Fig 6D), suggesting that most spine necks constitutively compartmentalize electrical signals in the head of spines.

We next estimated the elevation of calcium ion concentration induced in spine heads by an EPSP. The amplitude of calcium transients (Δ[Ca^2+^]) was similar in SiSs and DiSs and varied nonlinearly with A_ePSD_ (Fig 6E). A_ePSD_ accounted for 30% of Δ[Ca^2+^] in SiSs and 45% in DiSs, followed by R_neck_ (9%; S1 Table). NMDA receptors (NMDARs) had the largest contribution to Δ[Ca^2+^], consistent with numerous experimental observations reviewed in [125,126] (S9 Fig). Overall, our model provides quantitative insights into the variability of EPSP amplitude originating from spine diversity and highlights differences in the contribution of morphological parameters to membrane depolarization and calcium signals in SiSs and DiSs.

### Temporal interplay of excitatory and inhibitory signals in dually innervated spines

**Fig 7:**
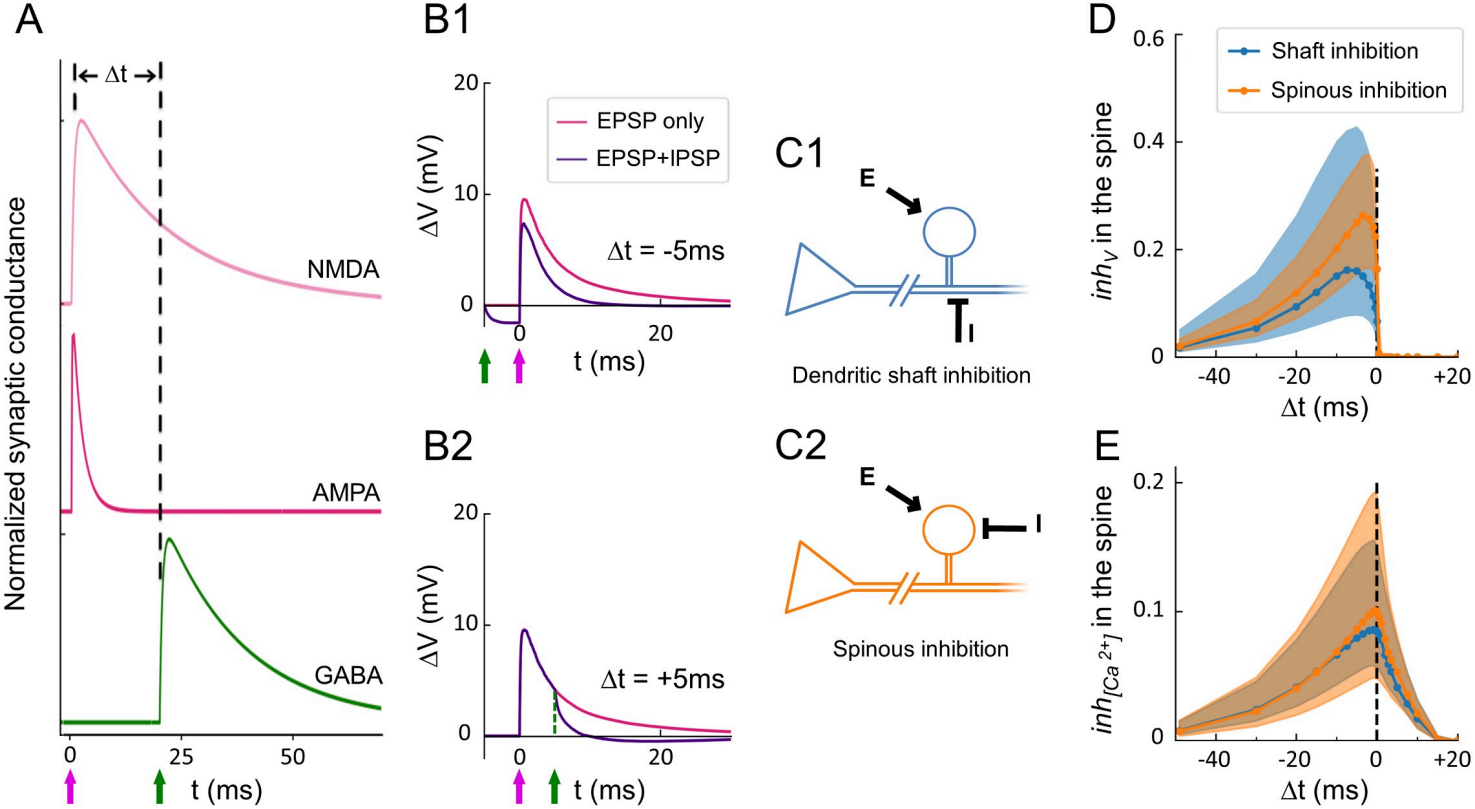
Input timing determines EPSP and IPSP integration and decoupling of voltage and calcium signals in DiSs. (A) Schematic: excitatory AMPA and NMDA conductances were activated at t = 0. The inhibitory GABAergic conductance was activated at an interval Δt before or after the onset of excitation. (B) Examples of the time course of depolarization in the spine head for Δt = +5 ms (B1) and Δt = −5 ms (B2) (purple curves) compared to no inhibition (magenta curves). Arrows represent the onset of excitatory and inhibitory inputs (magenta and green arrows, respectively). (C) Schematics: inhibition (┬ symbol) of an EPSP (arrow) by an IPSP elicited either in the shaft next to the spine (C1, blue) or directly in the spine head (C2, orange). (D) Voltage inhibition in the spine head, *inh*_*V*_, induced by dendritic (blue) or spinous (orange) IPSPs as a function of Δt. (E) Inhibition of the calcium influx in the spine head, *inh*_*[Ca*_*2+*_*]*_, induced by dendritic (blue) or spinous (orange) IPSPs as a function of Δt. The data underlying this figure can be found in https://www.opendata.bio.ens.psl.eu/3DCLEM-Spines/data/Data_related_to_Fig 7.xlsx (login: guest; password: EnsData0811). AMPA, α-amino-3-hydroxy-5-methyl-4-isoxazolepropionic acid; DiSs, dually innervated spines; EPSP, excitatory postsynaptic potential; IPSP, inhibitory postsynaptic potential; NMDA, N-Methyl-D-aspartate.

We used our model to compare the effects of dendritic shaft inhibition and spinous inhibition, whose functional relevance is still unclear. To understand how spine ultrastructure and iPSD location influence synaptic integration, we modeled the interaction between one IPSP and one EPSP under the constraint of our morphological measurements. Assessing the extent of signal variability originating from spine morphological heterogeneity required a large number of simulations (*N* ≥ 1,000). To expand our distribution of DiSs and shaft iPSDs (S1 Data), we used a bootstrapping method [127], which provided unbiased estimations of the mean and variance of the signals (see Materials and methods). We first simulated the interaction of one EPSP and one IPSP generated with a time difference of Δt (Fig 7A). For Δt < 0 (IPSP before EPSP), IPSPs decreased the amplitude of the EPSPs (Fig 7B1). For Δt > 0 (IPSP after EPSP), IPSPs had no effect on the amplitude but abruptly decreased the tail of the EPSPs [128] (Fig 7B2).

Next, we modeled *N =* 3,700 DiSs located between 20 μm and 140 μm from the soma, and we compared 2 configurations: (1) activation of the ePSD of the DiS and a shaft iPSD placed next to it (Δx = 0.7 μm) (Fig 7C1); and (2) activation of the ePSD and iPSD of the DiS (Fig 7C2). To quantify how the timing of inhibition affects EPSP amplitude, we compared the amplitude of individual EPSPs in the absence (ΔV_max,E_) or presence (ΔV_max,E+I_) of inhibition and computed the drop in depolarization amplitude *inh*_*V*_(Δt) = 1 − ΔV_max,E+I_ / ΔV_max,E_. *Inh*_*V*_ = 0 indicates no inhibition of the EPSP, while *inh*_*V*_ = 1 indicates complete inhibition of the EPSP. *Inh*_*V*_ was an asymmetrical function of Δt (Fig 7D) [36,128]. It was maximal at Δt = −4 ms for spinous inhibition and at Δt = −6 ms for dendritic shaft inhibition, and it decreased ΔV_max_ by 26% and 16%, respectively (median values at the peaks in Fig 7D). Therefore, despite the smaller size of spinous iPSDs compared to shaft iPSDs and the favorable placement of the shaft iPSD in these simulations, our results indicate that local spinous inhibition is stronger than shaft inhibition.

To determine how the timing of inhibition impacts calcium signals in DiSs, we then calculated *inh*_*[Ca*_*2+*_*]*_ (Δt) = 1 − [Ca^2+^]_max,E+I_ / [Ca^2+^]_max,E_. *Inh*_*[Ca*_*2+*_*]*_ peaked at Δt = 0 ms for both spinous and shaft inhibition. More precisely, spinous inhibition reduced calcium transient amplitude by 10% in average, reaching >36% in the top 10% simulations, while shaft inhibition reduced it by 8.6% in average and >28% in the top 10% simulations (Fig 7E), consistent with double uncaging experiments [129]. Importantly, IPSPs could decrease the amplitude of calcium transients within a short time window (Δt between 0 and +10 ms) in which depolarization amplitude was not affected (Fig 7D and 7E), thereby decoupling calcium signals from electrical activity in DiSs.

To further assess the importance of spine morphological features in the model outputs, we examined the case of adult L2/3 cortical PNs expressing the human-specific gene *Slit-Robo Rho GTPAse-activating protein 2C (SRGAP2C)*. We have previously shown that *SRGAP2C* regulates synapse density, spine neck length, and DiS proportion in oblique apical dendrites [97,130]. CLEM reconstructions showed that *SRGAP2C* expression did not affect the density of synapses or DiSs in basal dendrites. However, dendritic spines in the basal dendrites of *SRGAPC*-expressing neurons had smaller ePSDs and smaller necks than in control neurons (see panels A-C in S10 Fig). Remarkably, *SRGAP2C* expression slightly increased the impact of inhibition on ΔV_max_ in spine heads, while it strongly reduced the inhibition of Δ[Ca^2+^] (see panel D in S10 Fig), suggesting distinct voltage/calcium dynamics imposed by spine ultrastructure. These results highlight the critical need of morphologically constrained models to understand synaptic integration.

## Discussion

In the present study, we developed a novel 3D-CLEM workflow allowing the ultrastructural and quantitative characterization of specific populations of dendritic spines in genetically defined types of neurons. We used this workflow to exhaustively reconstruct spines and synaptic contacts along the basal dendrites of fluorescently labelled L2/3 cortical PNs of the SSC and to provide a quantitative description of their diversity. We input our measurements in a computational model to analyze the variability of electrical and calcium synaptic signals originating from spine ultrastructural diversity and to characterize the local integration of excitatory and inhibitory inputs in dendritic spines. Our results shed light on unique properties of DiSs, which represent 10% of all spines and 38% of all inhibitory synapses along the basal dendrites of L2/3 cortical PNs. While individual inhibitory synapses distributed along dendritic shafts can be powerful enough to block several EPSPs [102], we show that spinous inhibition affects excitatory signals more efficiently than shaft inhibition in DiSs. Furthermore, the activation of a spinous inhibitory synapse within a few milliseconds after an EPSP can decouple voltage and calcium signals in DiSs, which could impact calcium-dependent signaling cascades that drive spine plasticity.

The molecular composition and biophysical properties of spines and synapses are heterogeneous along dendritic segments and across dendritic trees. However, most computational models usually overlook this diversity and consider that inhibition only occurs on the soma or in dendrites (e.g., [131–133]). The 3D-CLEM approach we propose provides an accessible solution for detailed quantification of synaptic diversity beyond the micrometer scale in intact brain circuits. Although there can be variation in synaptic transmission at a given synapse due to the stochasticity of the molecular processes involved, EM measurements of synapse size are predictive of the average strength of individual synapses in L2/3 cortical PNs [110]. Therefore, our approach will help implement morphologically constrained computational models to improve modeling accuracy and assess the functional outcomes of ultrastructural changes induced by experience, mutations, or diseases. Our workflow is applicable to any type of tissue and allows anatomical measurements of any kind of genetically labelled cells and organelles. One technical limitation is the need for chemical fixation, which may distort tissue morphology [134,135] and require correction based on a morphological comparison with physically fixed tissues (see panel B3 in S7 Fig) in order to reliably depict *in vivo* situations. Future development of aldehyde-free cryo-CLEM methods for tissue analysis will be important to grant access to cellular and synaptic ultrastructure in close-to-native environments.

Applying 3D-CLEM to the basal dendrites of L2/3 cortical PNs allowed us to quantitatively describe the landscape of synaptic diversity and to characterize the ultrastructural features of a scarce population of dendritic spines receiving both excitatory and inhibitory synaptic inputs (DiSs). In the cortex, DiSs are mostly contacted by VGluT2-positive excitatory thalamocortical inputs [15], and they receive inhibition from somatostatin-expressing and parvalbumin-expressing interneurons [129,136], which are the 2 main sources of inhibitory inputs to the basal dendrites of L2/3 cortical PNs [137,138]. *In vivo* 2-photon imaging experiments have shown that DiSs are among the most stable spines along the dendrites of L2/3 PNs [103]. The inhibitory synapse in DiSs is smaller and more labile than inhibitory synapses along dendritic shafts, and it is very sensitive to sensory experience [73,93,94,103]. Whisker stimulation induces a lasting increase in the occurrence of iPDSs in spines of the barrel cortex [93], and monocular deprivation destabilizes iPSDs housed in spines of the visual cortex [73,94,103], suggesting their role in experience-dependent plasticity. Our morphological and computational analysis provides new insights into the biophysical properties of DiSs. We show that DiSs have larger heads and larger ePSDs than SiSs, and most often contain an SA. However, the ratio between mean spine neck diameter and spine head volume (or ePSD area) was smaller in DiSs than in SiSs, and DiSs had longer necks than SiSs of comparable head volume, so that EPSPs of similar amplitudes encounter a higher neck resistance in DiSs than in SiSs. Thus, DiSs are uniquely compartmentalized by their ultrastructural features and the presence of an inhibitory synapse.

Our model predicts that IPSPs occurring in DiSs within milliseconds after an EPSP can curtail calcium transients without affecting depolarization, thereby locally decoupling voltage and calcium signaling. This is expected to impact the induction of long-term forms of synaptic plasticity, such as long-term potentiation (LTP) or long-term depression (LTD), which underlie learning and memory [79,139–141]. The induction of LTP versus LTD is determined by the magnitude and time course of calcium flux, with brief, high calcium elevation generating LTP, sustained moderate calcium elevation generating LTD, and low calcium level inducing no plasticity [142–144]. Therefore, a small reduction in the amplitude of calcium transients may limit spine potentiation or even cause depression [145–147]. In the cortex, thalamocortical inputs may contact DiSs on the basal dendrites of L2/3 PNs both directly (excitatory connection) and indirectly through feed-forward inhibition via parvalbumin-expressing fast-spiking interneurons [148,149]. The delay between thalamocortical excitatory and feed-forward inhibitory signals is typically +1 ms to +3 ms [148], within the 10 ms time window for voltage–calcium decoupling in DiSs. Therefore, the presence of inhibitory synapses in DiSs could prevent synaptic potentiation and thereby increase the temporal precision of cortical response to sensory stimulation [93,148,150]. On the contrary, the removal of spine inhibitory synapses during experience-dependent plasticity such as monocular deprivation could favor synaptic potentiation to strengthen inputs from the nondeprived eye [73,94,151]. Likewise, the partial alleviation of calcium transient reduction by the human-specific gene *SRGAP2C* might facilitate long-term plasticity in DiSs. In the future, these hypotheses will have to be tested experimentally and using more elaborate morphologically constrained models allowing the computation of complex events at the basis of long-term plasticity.

Our understanding of synaptic and dendritic computations is intimately linked to the quantitative description of synaptic distribution, ultrastructure, nano-organization, activity, and diversity in neural circuits. The CLEM workflow we propose opens new avenues for the ultrastructural characterization of synapses with distinct molecular signatures defining their identity or activation profile. Another milestone to better model the biophysics of synaptic integration will be to combine EM with quantitative super-resolution LM to measure the density and nano-organization of molecular species (e.g., AMPARs, NMDARs, and VDCCs) in specific populations of synapses in intact brain circuits. Combining circuit and super-resolution approaches through CLEM will be critical to refine large-scale circuit models [74,152,153] (but see [32]) and bridge the gap between molecular, system, and computational neurosciences.

## Materials and methods

### Ethics statement

All animals were handled according to European and French regulations (directive 2010/63, decree 2013–118). The reference of our authorization was APAFIS#1530-2015082611508691v3. It was delivered by the French Ministry of Research after evaluation by the Comité d’Ethique en Experimentation Animale n°005.

### Animals and *in utero* cortical electroporation

*In utero* cortical electroporation was performed as described previously [154]. Briefly, pregnant Swiss female mice at E15.5 (Janvier Labs, France) were anesthetized with isoflurane (3.5% for induction, 2% during the surgery) and subcutaneously injected with 0.1 mg/kg of buprenorphine for analgesia. The uterine horns were exposed after laparotomy. Electroporation was performed using a square wave electroporator (ECM 830, BTX, Holliston, MA) and tweezer-type platinum disc electrodes (5-mm-diameter, Sonidel, Dublin, Ireland). The electroporation settings were 4 pulses of 40 V for 50 ms with 500 ms interval. Endotoxin-free DNA was injected using a glass pipette into one ventricle of the mouse embryos at the following concentrations: pH1SCV2 TdTomato: 0.5 μg/μL; pCAG EGFP-GPHN: 0.3 μg/μL; and pCAHA SRGAP2C: 0.7 μg/μL. All constructs have been described before [97].

### Cortical slice preparation

Electroporated animals aged between postnatal day P78 and P129 were anesthetized with ketamin 100 mg/kg and xylazin 10 mg/kg and intracardiacally perfused with first 0.1 mL of heparin (5000 U.I/mL, Sanofi, Paris, France), then an aqueous solution of 4% w/v paraformaldehyde (PFA) (CliniSciences) and 0.5% glutaraldehyde (GA) (Clinisciences, Nanterre, France) in 0.1 M phosphate-buffered saline (PBS). The fixative solution was made extemporaneously and kept at ice-cold temperature throughout the perfusion. The perfusion was gravity-driven at a flow rate of about 0.2 ml/s, and the total perfused volume was about 100 ml per animal. Brains were collected and postfixed overnight at 4°C in a 4% PFA solution. Coronal brain sections with 30-μm thickness were obtained using a vibrating microtome (Leica VT1200S).

### Fluorescence microscopy of fixed tissue

Slices containing electroporated neurons were trimmed to small (5 to 10 mm^2^) pieces centered on a relatively isolated fluorescent neuron, then mounted in a custom-made chamber on #1.5 glass coverslips. The mounting procedure consisted in enclosing the slices between the glass coverslip and the bottom of a cell culture insert (Falcon, ref. 353095) adapted to the flat surface with a silicon O-ring gasket (Leica, Wetzlar, Germany) and fixed with fast-curing silicon glue (see panel A in S1 Fig). Volumes of GFP and tdTomato signals were acquired in 12 bits mode (1024 × 1024 pixels) with z-steps of 400 nm using an inverted Leica TCS SP8 confocal laser scanning microscope equipped with a tunable white laser and hybrid detectors and controlled by the LAF AS software. The objective lenses were a 10X PlanApo, NA 0.45 lens for identifying electroporated neurons, and a 100X HC-PL APO, NA 1.44 CORR CS lens (Leica) for higher magnification images. GFP-GPHN puncta with a peak signal intensity at least 4 times above shot noise background levels were considered for CLEM.

### Placement of DAB fiducial landmarks

Following confocal imaging, slices were immersed in a solution of 1 mg/mL 3,3′-diaminobenzidine tetrahydrochloride (DAB, Sigma Aldrich, Darmstadt, Germany) in Tris buffer (0.05 M, pH 7.4). The plugin “LAS X FRAP” (Leica) was used to focus the pulsed laser in the tissue in custom patterns of 10-to-20 points using 100% power in 4 wavelengths (470 to 494 nm) for 30 seconds to 60 seconds per point at 3 different depths: the top of the slice, the depth of the targeted soma, then the bottom of the slice (surface closest to the objective). DAB precipitates were imaged in transmitted light mode. Slices were subsequently rinsed twice in Tris buffer and prepared for EM.

### Tissue preparation for serial block–face scanning electron microscopy (SBEM)

Using a scalpel blade under a M165FC stereomicroscope (Leica), imaged tissue slices were cut to approximately 1 mm^2^ asymmetrical pieces of tissue centered on the ROI, and then kept in plastic baskets (Leica) through the osmification and dehydration steps. Samples were treated using an osmium bridging technique adapted from the NCMIR protocol (OTO) [155]. The samples were washed 3 times in ddH_2_O and immersed for 1 hour in a reduced osmium solution containing 2% osmium tetroxide and 1.5% potassium ferrocyanide in ddH_2_O. Samples were then immersed for 20 minutes in a 1% thiocarbohydrazide (TCH) solution (EMS, Hatfield, PA) prepared in ddH_2_O at room temperature. The samples were then postfixed with 2% OsO_4_ in ddH_2_O for 30 minutes at room temperature and colored *en bloc* with 1% aqueous uranyl acetate at 4°C during 12 hours. Postfixed samples were subjected to Walton’s *en bloc* lead aspartate staining at 60°C for 30 minutes (Walton, 1979). After dehydration in graded concentrations of ice-cold ethanol solutions (20%, 50%, 70%, 90%, and twice 100%, 5 minutes per step), the samples were rinsed twice for 10 minutes in ice-cold anhydrous acetone. Samples were then infiltrated at room temperature with graded concentrations of Durcupan (EMS) prepared without plastifier (components A, B, and C only). In detail, blocks were infiltrated with 25% Durcupan for 30 minutes, 50% Durcupan for 30 minutes, 75% Durcupan for 2 hours, 100% Durcupan overnight, and 100% fresh Durcupan for 2 hours before being polymerized in a minimal amount of resin in a flat orientation in a sandwich of ACLAR 33C Films (EMS) at 60°C for 48 hours. Samples were mounted on aluminum pins using conductive colloidal silver glue (EMS). Before curing, tissue blocks were pressed parallel to the pin surface using a modified glass knife with 0° clearance angle on an ultramicrotome (Ultracut UC7, Leica) in order to minimize the angular mismatch between LM and SEM imaging planes. Pins then cured overnight at 60°C. Samples were then trimmed around the ROI with the help of fluorescent overviews of the ROI within their asymmetrical shape. Minimal surfacing ensured that superficial DAB landmarks were detected at the SBEM before block-facing.

### SBEM acquisition

SBEM imaging was performed with a Teneo VS microscope (FEI, Eindhoven, The Netherlands) on the ImagoSeine imaging platform at Institut Jacques Monod, Paris. The software MAPS (Thermo Fisher Scientific, Waltham, MA) was used to acquire SEM images of targeted volumes at various magnifications. Acquisition parameters were 1,7830 kV, 500 ns/px, 100 pA, 40-nm-thick sectioning, and 8200 × 8200 pixels resolution with either 2.5 nm or 25 nm pixel size for high- and low-magnification images, respectively. Placing an electromagnetic trap above the diamond knife to catch discarded tissue sections during days-long imaging sessions was instrumental to achieve continuous 3D-EM acquisitions.

### Image segmentation

Dendrites were segmented from SBEM stacks using the software Microscopy Image Browser (MIB) [156]. 3D reconstruction was performed with the software IMOD [157] (http://bio3d.colorado.edu/imod/). Individual PSDs were manually outlined based on the apposition with a presynaptic terminal and differences in membrane contrast and thickness between postsynaptic and nonsynaptic membranes on individual SBEM images (see example movie at https://www.opendata.bio.ens.psl.eu/3DCLEM-Spines/S1_Movie.zip; login: guest, password: EnsData0811). 3D spine models were imported in the software Blender (www.blender.org) for subsampling, and the quantification of spine section areas along their main axis was done with in-house python scripts. Other measurements were performed using IMOD and in-house python scripts. Models and raw data are accessible at https://www.opendata.bio.ens.psl.eu/3DCLEM-Spines (login: guest; password: EnsData0811).

### Tissue preparation for tissue shrinkage estimation

Two female mice (21 days postnatal) were used for the analysis of tissue shrinkage induced by chemical fixation. Mice were decapitated, and their brains were rapidly removed. The brains were transferred to an ice-cold dissection medium, containing (in mM) KCl, 2.5; NaHCO_3_, 25; NaH_2_PO_4_, 1; MgSO_4_, 8; glucose, 10 (pH 7.4). A mix of 95% O_2_ and 5% CO_2_ was bubbled through the medium for 30 minutes before use. Coronal brain sections with 300-μm thickness were obtained using a vibrating microtome (Leica VT1200S). Small fragments of the SSC were cut from those slices and fixed either by immersion in an ice-cold PBS solution containing 4% PFA and 0.5% GA or in frozen with liquid nitrogen under a pressure of 2,100 bars using a high-pressure freezing (HPF) system (HPM100, Leica). For HPF-frozen samples, the interval between removal of the brain and vitrification was about 7 minutes. Cryo-substitution and tissue embedding were performed in a Reichert AFS apparatus (Leica). Cryo-substitution was performed in acetone containing 0.1% tannic acid at −90°C for 4 days with one change of solution, then in acetone containing 2% osmium during the last 7 hours at −90°C. Samples were thawed slowly (5°C/h) to −20°C and maintained at −20°C for 16 additional hours, then thawed to 4°C (10°C/h). At 4°C, the slices were immediately washed in pure acetone. Samples were rinsed several times in acetone, then warmed to room temperature and incubated in 50% acetone–50% araldite epoxy resin for 1 hour, followed by 10% acetone–90% araldite for 2 hours. Samples were then incubated twice in araldite for 2 hours before hardening at 60°C for 48 hours. As for chemically fixed sections, they were postfixed for 30 minutes in ice-cold 2% osmium solution, rinsed in PBS buffer, dehydrated in graded ice-cold ethanol solutions, and rinsed twice in ice-cold acetone, before undergoing the same resin infiltration and embedding steps as HPF-frozen samples. After embedding, ultrathin sections were cut in L2/3 of the SSC, orthogonally to the apical dendrites of PNs, 200 to 300 μm from the pial surface using an ultramicrotome (Ultracut UC7, Leica). Ultrathin (pale yellow) sections were collected on formwar-coated nickel slot grids, then counterstained with 5% uranyl acetate in 70% methanol for 10 minutes, washed in distilled water, and air dried before observation on a Philips TECNAI 12 electron microscope (Thermo Fisher Scientific).

### Measurement of shrinkage correction factors

Ultrathin sections of both HPF-frozen tissues and chemically fixed tissues were observed using a Philips TECNAI 12 electron microscope (Thermo Fisher Scientific). Cellular compartments contacted by a presynaptic bouton containing synaptic vesicles and exhibiting a visible electron-dense PSD at the contact site, but no mitochondrion within their cytosol, were identified as dendritic spine heads. Cross-section areas of random spine heads and the curvilinear lengths of their PSD were quantified in both conditions using the softwares MIB and IMOD. *N =* 277 spine head sections were segmented in HPF-frozen cortical slices from 2 female mice, and *N* = 371 spine head sections were segmented in chemically fixed cortical slices originating from the same 2 mice. χ^2^ minimization was used between spine head cross-section area distributions in HPF or OTO conditions to compute average volume shrinkage and correction factors. PSD areas were not corrected as they exhibited no shrinkage.

### Computation of the diffusional neck resistance

The diffusional resistance of spine necks W_neck_ was measured as follows. Using IMOD, we first modeled in 3D the principal axis of each spine neck as an open contour of total length L_axis_ connecting the base of the neck to the base of the spine head. Using Blender, we interpolated each spine neck path linearly with 100 points. We named P(ℓ) the plane that bisected the spine neck model orthogonally to the path at the abscissa ℓ, and A(ℓ) the spine neck cross-section within P(ℓ). In spines containing an SA, we corrected A(ℓ) by a scaling factor β(ℓ) = 1 − (D_SA_/D_spine_)^2^(ℓ), where D_SA_/D_spine_(ℓ) is the local ratio of SA and neck diameter. We measured D_SA_/D_spine_ orthogonally to the neck path in 10 SA+ spines and in 3 different locations per spine on SBEM images: at the spine stem (ℓ/L_axis_ = 0.1), at the center of the spine neck (ℓ/L_axis_ = 0.5), and at the stem of the head (ℓ/L_axis_ = 0.9). D_SA_/D_spine_ was 44% ± 11%, 31% ± 8%, and 37% ± 8% respectively, and fluctuations were not statistically significant. We then divided each SA+ spine neck in thirds and scaled their neck cross-section areas along neck axis A_SA+_(ℓ) = β(ℓ)A(ℓ) before computing W_neck_ = ∫ dℓ / A(ℓ) for all spines, using Simpson’s integration rule.

### Multicompartment electrical model

All simulations were implemented in Python using NEURON libraries [158] and in-house scripts. Ordinary differential equations were solved with NEURON-default backward Euler method, with Δt = 0.05 ms. Scripts and model definition files are available in a GitHub repository: https://github.com/p-serna/SpineModel. Biophysical constants were taken from the literature as follows: membrane capacitance C_m_ = 1 μF/cm^2^ [38]; cytosolic resistivity ρ = 300 Ω.cm [84,159]; synaptic conductivities were modeled as biexponential functions g(t) = A g_max_ (e^- t / t 2^ − e^- t / t 1^) where A is a normalizing constant and (t_1_, t_2_) define the kinetics of the synapses: GABAergic conductance (t_1_, t_2_) = (0.5, 15) ms, AMPAR-dependent conductance (t_1_, t_2_) = (0.1, 1.8) ms, NMDAR-dependent conductance (t_1_, t_2_) = (0.5, 17.0) ms (ModelDB: https://senselab.med.yale.edu/ModelDB/). The magnesium block of NMDA receptors was modeled by a voltage-dependent factor [160]. Remaining free parameters comprised the following: the leaking conductivity g_m_ (or, equivalently, the membrane time constant T_m_); the peak synaptic conductance per area: g_AMPA_, g_NMDA_, g_GABA_; and the total membrane area of the modeled neuron. These parameters were adjusted so that signal distributions fitted published electrophysiological recordings [114,115,118,119,121]. In more detail, we first set up one “ball-and-stick” model per segmented spine (*N =* 390). The dendrite hosting the modeled spine was generated as a tube of diameter d_dendrite_ = 0.87 μm, and length L_dendrite_ = 140 μm. This dendrite was split in 3 segments, and the modeled spine was placed on the 2-μm-long middle segment. To account for the passive electrical effects of neighboring spines, the membrane surfaces of both the proximal and distal sections of the studied dendrite were scaled by a correction factor γ = 1 + <A_spine_> d_spine_ / π d_dendrite_ = 3.34, with the density d_spine_ = 1.63 spine.μm^−1^ and the average spine membrane area <A_spine_> = 3.89 μm^2^. Spine head shrinkage was corrected by scaling the length and diameter of all spine heads by a factor S = 1.22 and spine head volume by S^3^ = 1.81. PSD areas were not scaled (see panel C3 in S7 Fig). The leakage resistance was fitted to 65 MΩ [120], yielding a total membrane surface of the modeled neurons: A_mb,total_ = 18,550 μm^2^. We calibrated synaptic conductances type by type, by fitting the signals generated in the whole distribution of 390 constrained models to published electrophysiological recordings. AMPA conductances of all excitatory synapses were set proportional to ePSD area and scaled by the free parameter g_A_. In each model, we activated the AMPAR component of excitatory synapses and monitored the amplitude of resulting EPSCs in the soma. The average EPSC amplitude was adjusted to 58 pA [115,119], yielding a scaling factor g_A_ = 3.15 nS/μm^2^, which takes into account the average number of excitatory contacts per axon per PN in L2/3 of mouse SSC: N_ePSD/axon_ = 2.8 [115]. After this calibration, the maximal AMPA synaptic conductance g_AMPA_ ranged from 0.04 nS to 3.13 nS (0.456 ± 0.434 nS). NMDA conductances of all excitatory synapses were set proportional to ePSD area and scaled by the free parameter g_N_. In each model, we activated both NMDA and AMPA components of excitatory synapses and fitted the amplitude ratio between the average AMPA+NMDA and AMPA-only responses to 1.05 [119], yielding g_N_ = 3.4 nS/μm^2^. As a result, g_NMDA_ ranged from 0.04 nS to 3.42 nS (0.498 ± 0.474 nS), in line with the literature [120]. Voltage-dependent sodium and potassium conductances were not included in our model because the amplitude of single EPSPs remained subthreshold (<1% of synapses generate EPSPs large enough to activate these conductances [74,161]; see Fig 6B and 6C). Therefore, this model is not fit to compute the integration of multiples EPSPs or spikes. Considering inhibition, the GABA conductances of all inhibitory synapses were set proportional to iPSD area and scaled by the free parameter g_G_ = 5.9 nS/μm^2^ to adjust the mean conductance of dendritic inhibitory synapses to 1 nS [37,121,122]. Following this calibration, g_GABA_ ranged from 0.33 nS to 3.36 nS (1.00 ± 0.577 nS) for synapses located on the shaft and from 0.19 nS to 1.56 nS (0.528 ± 0.277 nS) for inhibitory synapses located on spines. The reversal potential of chloride ions (E_Cl_-) was set to −80 mV [162] and considered constant, as it is regulated on timescales exceeding 100 ms [163], and we modeled signals in the 10 ms timescale. Calcium influxes were modeled in spine heads as output of the opening of NMDARs and VDCCs. The dynamics of L-, N-, and Q-type VDCCs were obtained from ModelDB (accession n°: 151458), and their conductivities were scaled to the head membrane area of each spine, A_head_, excluding synaptic area(s). VDCC-type ratios and calcium conductivities were adjusted by fitting the average amplitude of calcium concentration transients to 20% of the NMDA conductance [112]. Calcium uptake from cytosolic buffers was set to 95% to yield an average amplitude of Ca^2+^ concentration transients of 0.7 μM [113].

### Bootstrapping

To simulate a large number of spine–spine interactions with limited redundancy, we expanded our distribution of spines using a “smooth” bootstrapping method [127]. Specifically, the dataset (i.e., a matrix of dimensions N × N_f_) was resampled to generate a new matrix of dimension M × N_f_, where N is the number of spines, N_f_ is the number of selected features, and M is the final number of synthetic spines. M rows were randomly selected in the original dataset, and zero-centered, feature-dependent Gaussian noise was added to each element of the matrix (excluding absolute quantities, e.g., number of PSDs or presence of SA). To determine appropriate noise amplitude for each parameter, we generated a synthetic set of M = 500 spines from the original dataset, including Gaussian noise with an arbitrary amplitude σ on 1 parameter at a time. This new feature distribution was compared to the original distribution using a 2-sample Kolmogorov–Smirnov test (KS test), and this procedure was repeated 1,000 times for each set value of σ. A conservative noise level (σ = 10%) was sufficient to smear parameter distributions while the fraction of synthetic sets that were statistically different from the original set (*p* < 0.05, KS test) remained 0 over 1,000 iterations. σ = 10% was valid for all relevant features, and we assumed that such a small noise amplitude would minimally interfere with nonlinear correlations in our dataset. Synthetically generated spines were then used to simulate elementary synaptic signaling using in-house python scripts. We also used bootstrapping to estimate standard deviations in our simulations.

### Statistics

No statistical methods were used to predetermine sample size. We used a one-way ANOVA on our 4 datasets to test that interneuron and intermice variability were small enough to pool all datasets together (S1 Data). We used KS test to determine that all measured morphological parameters followed a log-normal distribution (S1 Data). We used Mann–Whitney *U* test for statistical analyses of morphological parameters, except when comparing the probability for SiSs and DiSs to harbor SA, for which we used Pearson χ^2^ test. All results in the text are mean ± SD. In Figs 6 and 7, we plot medians as solid lines, as they better describe where log-normal distributions peak. Shaded areas represent 68% confidence intervals, which span approximately 1 SD on each side of the mean.

## Supporting information

S1 Fig ROI landmarking strategy for 3D-CLEM using DAB and a detachable chamber. (A) Schematic of ROI landmarking using DAB photo-oxidation. The tissue slice is held against a glass coverslip in a solution of DAB using a detachable chamber. Confocal imaging and ROI landmarking are performed using the same microscopy setup. (B) Transmitted light image of a cortical slice labelled with DAB precipitates. Varying the duration of UV illumination (T_ill_) allows adjusting DAB spot size. (C) Example of labeling pattern around an optically isolated fluorescent neuron. DAB was photoprecipitated by focusing UV light for 40 seconds at each highlighted location (pink dots in yellow circles). (D) Transmitted light image of the same field of view after DAB photoprecipitation. (E) Overlay showing DAB precipitates in D (dark spots) arranged similarly to the UV focusing pattern in C. (F) Schematic of slice retrieval after ROI landmarking. Detaching the chamber (wide arrow) allows taking the sample to EM preparation steps, i.e., Osmium–TCH–Osmium postfixation [155], dehydration, resin infiltration, and plastic embedding. (G) ROI recovery in SBEM. DAB precipitates (circles) generated at the surface of the sample mark the (x,y) coordinates of the ROI. They are detected with an electron beam (e−) before block facing the sample and acquiring SBEM images in targeted volumes. The DAB pattern generated at the depth of the targeted cell (in red) allows its retrospective identification. DAB, 3,3-diaminobenzidine; EM, electron microscopy; ROI, region of interest; SBEM, serial block–face scanning EM; TCH, thiocarbohydrazide; 3D-CLEM, 3D correlative light–electron microscopy.
(TIF)

S2 Fig Distance between spine and soma does not correlate with other measured parameters. Distance between spine and soma as a function of all measured morphological parameters of the spines that were segmented. No parameter exhibited a linear correlation with the distance between spine and soma. The data underlying this figure can be found in https://www.opendata.bio.ens.psl.eu/3DCLEM-Spines/data/Data_related_to_FigS2.xlsx (login: guest; password: EnsData0811). ePSD, excitatory postsynaptic density; iPSD, inhibitory PSD.
(TIF)

S3 Fig Effect of the SA on diffusional neck resistance. Distribution of the diffusional neck resistance (W_neck_) calculated using neck morphology for spines devoid of apparatus (SA−) or containing an SA (SA+). “SA+ uncorr.”: W_neck_ without SA correction for SA+ spines (*p* = 0.1 compared to corrected W_neck_). ***p < 0.001 calculated using Mann–Whitney test. The data underlying this figure can be found in https://www.opendata.bio.ens.psl.eu/3DCLEM-Spines/data/Data_related_to_FigS3.xlsx (login: guest; password: EnsData0811). n.s., not significant; SA, spine apparatus.
(TIF)

S4 Fig Distribution of inhibitory synapses along basal dendrites of L2/3 and L5 cortical PNs. (A) Representative segments of dendrites of L2/3 and L5 PNs expressing GFP-GPHN (green) and TdTomato (red) in juvenile (postnatal days P21–P27) or young (P10) mice. Neuronal progenitors of L2/3 and L5 PNs were electroporated *in utero* at E15.5 and E12.5, respectively. Scale bar: 4 μm. (B, C) Quantification of gephyrin cluster density (B) and proportion of gephyrin clusters in spines (C). Data were acquired and analyzed as in our previous work [97]. Gephyrin cluster distribution was similar in adult (data in main text) and juvenile mice (density: 3.5 ± 1.1 clusters per 10 μm of dendrite in adults and 4.5 ± 1.0 clusters per 10 μm of dendrite in juveniles; proportion of gephyrin clusters in spines: 38% and 36% in adults and juveniles, respectively). However, both the density of gephyrin clusters and their proportion in spines was lower in younger (P10) mice, suggesting that DiSs represent mature spines (see also [103]). The proportion of gephyrin clusters in spines was also lower in L5 PNs than in L2/3 neurons. N_L2/3 Juvenile_ = 18, N_L2/3 Young_ = 28, N_L5 Juvenile_ = 19. N represents the number of cells. Cells come from at least 3 animals per conditions. ***p < 0.001, *p < 0.05, ANOVA test followed by Tukey multiple comparisons test. The data underlying this figure can be found in https://www.opendata.bio.ens.psl.eu/3DCLEM-Spines/data/Data_related_to_FigS4.xlsx (login: guest; password: EnsData0811). DiSs, dually innervated spines; GFP-GPHN, GFP-tagged gephyrin; L2/3, layer 2/3; L5, layer 5; PN, pyramidal neuron.
(TIF)

S5 Fig Comparison of SiS and DiS neck diameter. Quantification of minimal neck diameter (A) and mean neck diameter (B) for all spines (*N =* 349 SiSs and 37 DiSs; solid lines) and for spines with V_head_ > 0.05 μm^3^ (*N* = 186 SiSs and 34 DiSs; dashed lines). *p* > 0.05 calculated using Mann–Whitney tests. The data underlying this figure can be found in https://www.opendata.bio.ens.psl.eu/3DCLEM-Spines/data/Data_related_to_FigS5.xlsx (login: guest; password: EnsData0811). DiSs, dually innervated spines; SiSs, singly innervated spines.
(TIF)

S6 Fig iPSD area as a function of DiS head volume. iPSD area as a function of spine head volume for *N* = 37 DiSs. Linear regression: R^2^ < 0.1. The data underlying this figure can be found in https://www.opendata.bio.ens.psl.eu/3DCLEM-Spines/data/Data_related_to_FigS6.xlsx (login: guest; password: EnsData0811). DiS, dually innervated spine; iPSD, inhibitory PSD.
(TIF)

S7 Fig Fixation-induced shrinkage of spine heads but not PSD area. (A) TEM images of L2/3 SSC acute slices from the same mouse (postnatal day P21) upon either chemical fixation with aldehydes (A1) or physical fixation with HPF (A2). Spine head section areas (indicated in red in A1 and in light blue in A2) and lengths of PSDs were segmented for quantification. Scale bars: 1 μm. (B) Normalized histograms of spine head cross-section areas for chemically fixed (Chem) samples (*N =* 194 for mouse “s1” and 178 for mouse “s2”; *p* = 0.13) or HPF samples (*N* = 128 for s1 and 150 for s2; *p* = 0.052) in B1 and B2, respectively. Data from mice s1 and s2 displayed no statistical difference and were pooled together in B3 to compare area distribution between HPF (blue) and chemically fixed tissue (orange). Head cross-section areas were 34% ± 5% smaller in chemically fixed samples (orange) than in HPF samples (blue), implying approximately 45% head volume shrinkage (*p* < 10^−8^). (C) Normalized histograms of PSD section lengths for chemically fixed (*p* = 0.44) and HPF samples (*p* = 0.17) in C1 and C2, respectively. PSDs were not significantly deformed by chemical fixation (C3; *p* = 0.10). Only significant (*p* < 0.05) *p*-values are shown. ***p < 0.001 calculated using Mann–Whitney test. The data underlying this figure can be found in https://www.opendata.bio.ens.psl.eu/3DCLEM-Spines/data/Data_related_to_FigS7.xlsx (login: guest; password: EnsData0811). HPF, high-pressure freezing; L2/3, layer 2/3; PSD, postsynaptic density; SSC, somatosensory cortex; TEM, transmission electron microscopy.
(TIF)

S8 Fig Influence of R_neck_ on ΔV_max_ in the spine head and dendritic shaft. Plot of the EPSP amplitude ΔV_max_ elicited in a dendritic spine while varying its neck resistance (R_neck_) and keeping all other parameters constant. Increasing R_neck_ causes ΔV_max_ to increase in the spine head (blue) and to decrease in the dendritic shaft (orange). The data underlying this figure can be found in https://www.opendata.bio.ens.psl.eu/3DCLEM-Spines/data/Data_related_to_FigS8.xlsx (login: guest; password: EnsData0811). EPSP, excitatory postsynaptic potential.
(TIF)

S9 Fig Contribution of VDCCs and NMDAR to calcium transients. (A) Typical time course of a calcium transient elicited in spine head. The calcium signal was induced by an EPSP at t = 120 ms. The contributions of VDCCs and NMDARs to the total elevation of calcium concentration (blue curve) are plotted in orange and green, respectively. (B) Contribution of VDCCs to the total elevation of calcium concentration as a function of the estimated amplitude of the calcium transient in all spines (*N* = 390). The data underlying this figure can be found in https://www.opendata.bio.ens.psl.eu/3DCLEM-Spines/data/Data_related_to_FigS9.xlsx (login: guest; password: EnsData0811). EPSP, excitatory postsynaptic potential; NMDAR, NMDA receptor; VDCC, voltage-dependent calcium channel.
(TIF)

S10 Fig Morphofunctional properties of spines in basal dendrites of L2/3 neurons expressing the human-specific gene *SRGAP2C*. (A) Z-projection of the confocal stack showing a segment of basal dendrite of an L2/3 PN expressing cytosolic TdTomato (in magenta), GFP-GPHN (in green), and *SRGAP2C* in the adult mouse SSC. Neurons were electroporated *in utero* at E15.5. Numbers identify individual spines. Scale bar: 1 μm. (B) 3D-EM reconstruction. Individual dendritic spines are manually segmented and randomly colored. Spines that were detected in CLEM but not in LM alone are labelled in red. Scale bar: 1 μm. (C) Quantification of spine head volume (C1), ePSD area (C2), DiS–iPSD area (C3), neck length (L_neck_) (C4), mean neck diameter (C5), and the diffusional neck resistance (W_neck_) (C6) for WT spines (blue) and spines expressing SRGAP2C (red). *N* = 73 spines expressing SRGAP2C and 390 WT spines in panels C1–C2 and C4–C6. *N* = 6 DiSs expressing SRGAP2C and 37 WT DiSs in panel C3. ***p < 10^−3^ for all quantities except for iPSD area, Mann–Whitney test. Comparison with our previous work on oblique apical dendrites [97,127] suggests that *SRGAP2C* expression has dendrite-type specific consequences on spine density and morphology. (D) Similar panels as in Fig 7 but for spines along the basal dendrites of neurons expressing *SRGAP2C*. (D1) Voltage inhibition in the spine head, *inh*_*V*_, induced by dendritic (blue) or spinous (orange) IPSPs as a function of Δt. (D2) Inhibition of the calcium influx in the spine head, *inh*_*[Ca*_*2+*_*]*_, induced by dendritic (blue) or spinous (orange) IPSPs as a function of Δt. The inhibition of calcium signals was approximately 40% lower in neurons expressing *SRGAP2C* than in control neurons (Fig 7). The data underlying this figure can be found in https://www.opendata.bio.ens.psl.eu/3DCLEM-Spines/data/Data_related_to_FigS10.xlsx (login: guest; password: EnsData0811). CLEM, correlative light–electron microscopy; DiS, dually innervated spine; ePSD, excitatory postsynaptic density; GFP-GPHN, GFP-tagged gephyrin; iPSD, inhibitory PSD; IPSP, inhibitory postsynaptic potential; LM, light microscopy; L2/3, layer 2/3; PN, pyramidal neuron; SRGAP2C, Slit-Robo Rho GTPAse-activating protein 2C; SSC, somatosensory cortex; WT, wild-type; 3D-EM, three-dimensional electron microscopy.
(TIF)

S1 Table Contribution of morphological parameters to the variance of ΔV_max_ and Δ[Ca^2+^]_max_. Highest-ranking input parameters (A_ePSD_, R_neck_, and L_dend_) are sorted by decreasing contribution to the variance of the simulation outputs, as estimated with a GLM. Numbers indicate to which proportion input variables accounted for the variance of considered output.
(XLSX)

S1 Data Quantification of the morphology of spines and synapses. The first sheet of the .xlsx file describes the samples used in our experiments. The second sheet reports scores for log-normality and intersample variability tests for each morphological variable. The 2 last sheets report spine and PSD anatomy in all segmented dendrites. Legends are included in the rightmost column.
(XLSX)

## Abbreviations

AMPA: α-amino-3-hydroxy-5-methyl-4-isoxazolepropionic acid
DAB: 3,3-diaminobenzidine
DiSs: dually innervated spines
EM: electron microscopy
ePSD: excitatory postsynaptic density
EPSP: excitatory postsynaptic potential
GFP-GPHN: GFP-tagged gephyrin
GLM: generalized linear model
HPF: high-pressure freezing
iPSD: inhibitory postsynaptic density
IPSP: inhibitory postsynaptic potential
IUE: *in utero* electroporation
KS test: Kolmogorov–Smirnov test
LM: light microscopy
LTD: long-term depression
LTP: long-term potentiation
L2/3: layer 2/3
MIB: Microscopy Image Browser
NIRB: near-infrared branding
NMDA: N-Methyl-D-aspartate
PBS: phosphate-buffered saline
PFA: paraformaldehyde
PN: pyramidal neuron
RC: resistor–capacitor
ROI: region of interest
SA: spine apparatus
SBEM: serial block–face scanning EM
SER: smooth endoplasmic reticulum
SiSs: singly innervated spines
SRGAP2C: Slit-Robo Rho GTPAse-activating protein 2C
SSC: somatosensory cortex
STED: stimulated emission depletion
TCH: thiocarbohydrazide
VDCC: voltage-dependent calcium channel
3D-CLEM: 3D correlative light–electron microscopy
