# Supplementary figures and images for: Unique properties of dually innervated dendritic spines in pyramidal neurons of the somatosensory cortex uncovered by 3D correlative light and electron microscopy
Source: PLoS Biol. 2021 Aug 24;19(8):e3001375. doi: 10.1371/journal.pbio.3001375 (PMC8415616; doi:10.1371/journal.pbio.3001375)

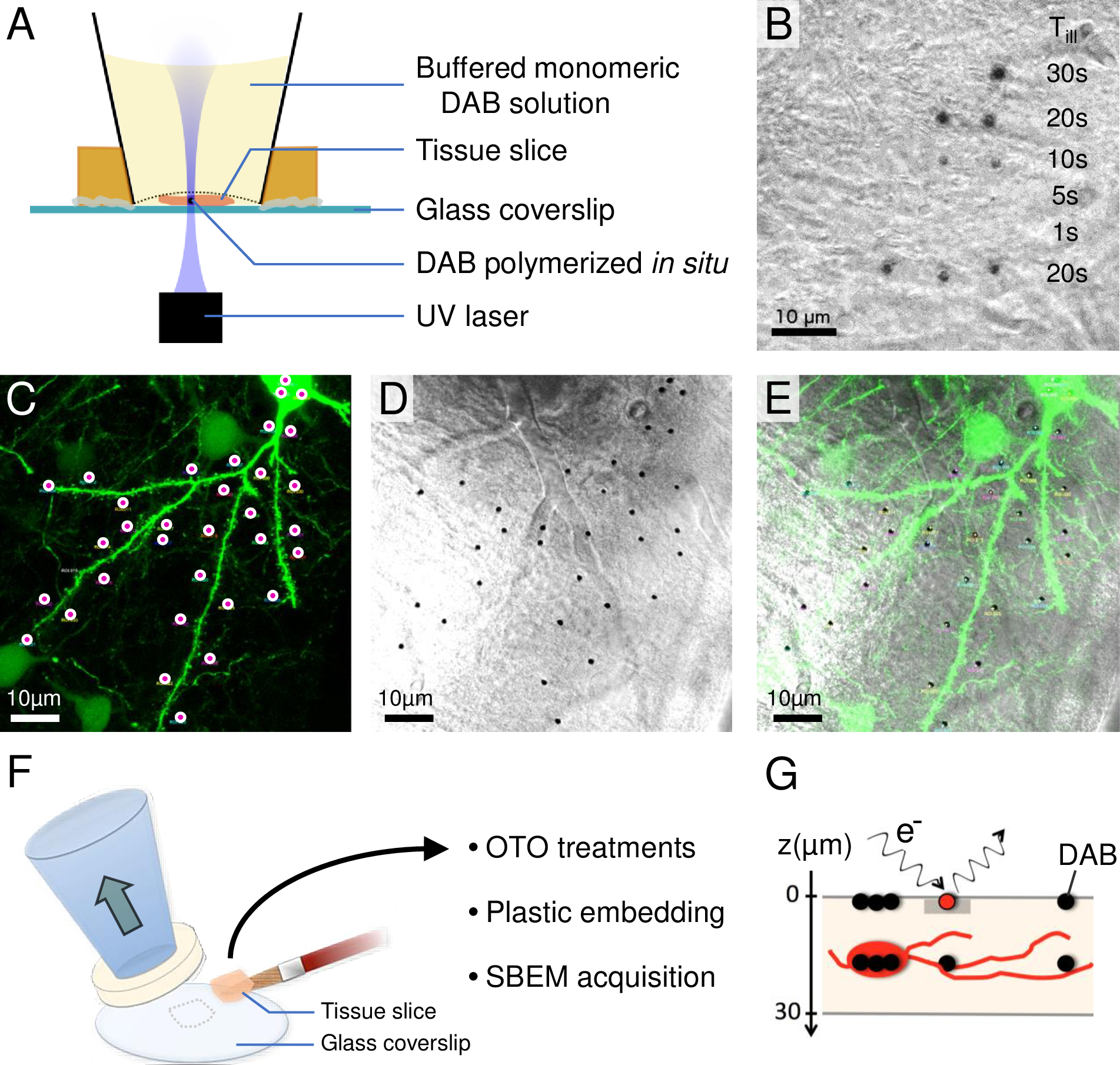

Supplement: S1 Fig — (A) Schematic of ROI landmarking using DAB photo-oxidation. The tissue slice is held against a glass coverslip in a solution of DAB using a detachable chamber. Confocal imaging and ROI landmarking are performed using the same microscopy setup. (B) Transmitted light image of a cortical slice labelled with DAB precipitates. Varying the duration of UV illumination (Till) allows adjusting DAB spot size. (C) Example of labeling pattern around an optically isolated fluorescent neuron. DAB was photoprecipitated by focusing UV light for 40 seconds at each highlighted location (pink dots in yellow circles). (D) Transmitted light image of the same field of view after DAB photoprecipitation. (E) Overlay showing DAB precipitates in D (dark spots) arranged similarly to the UV focusing pattern in C. (F) Schematic of slice retrieval after ROI landmarking. Detaching the chamber (wide arrow) allows taking the sample to EM preparation steps, i.e., Osmium–TCH–Osmium postfixation [155], dehydration, resin infiltration, and plastic embedding. (G) ROI recovery in SBEM. DAB precipitates (circles) generated at the surface of the sample mark the (x,y) coordinates of the ROI. They are detected with an electron beam (e−) before block facing the sample and acquiring SBEM images in targeted volumes. The DAB pattern generated at the depth of the targeted cell (in red) allows its retrospective identification. DAB, 3,3-diaminobenzidine; EM, electron microscopy; ROI, region of interest; SBEM, serial block–face scanning EM; TCH, thiocarbohydrazide; 3D-CLEM, 3D correlative light–electron microscopy. (TIF) [file pbio.3001375.s001.tif]

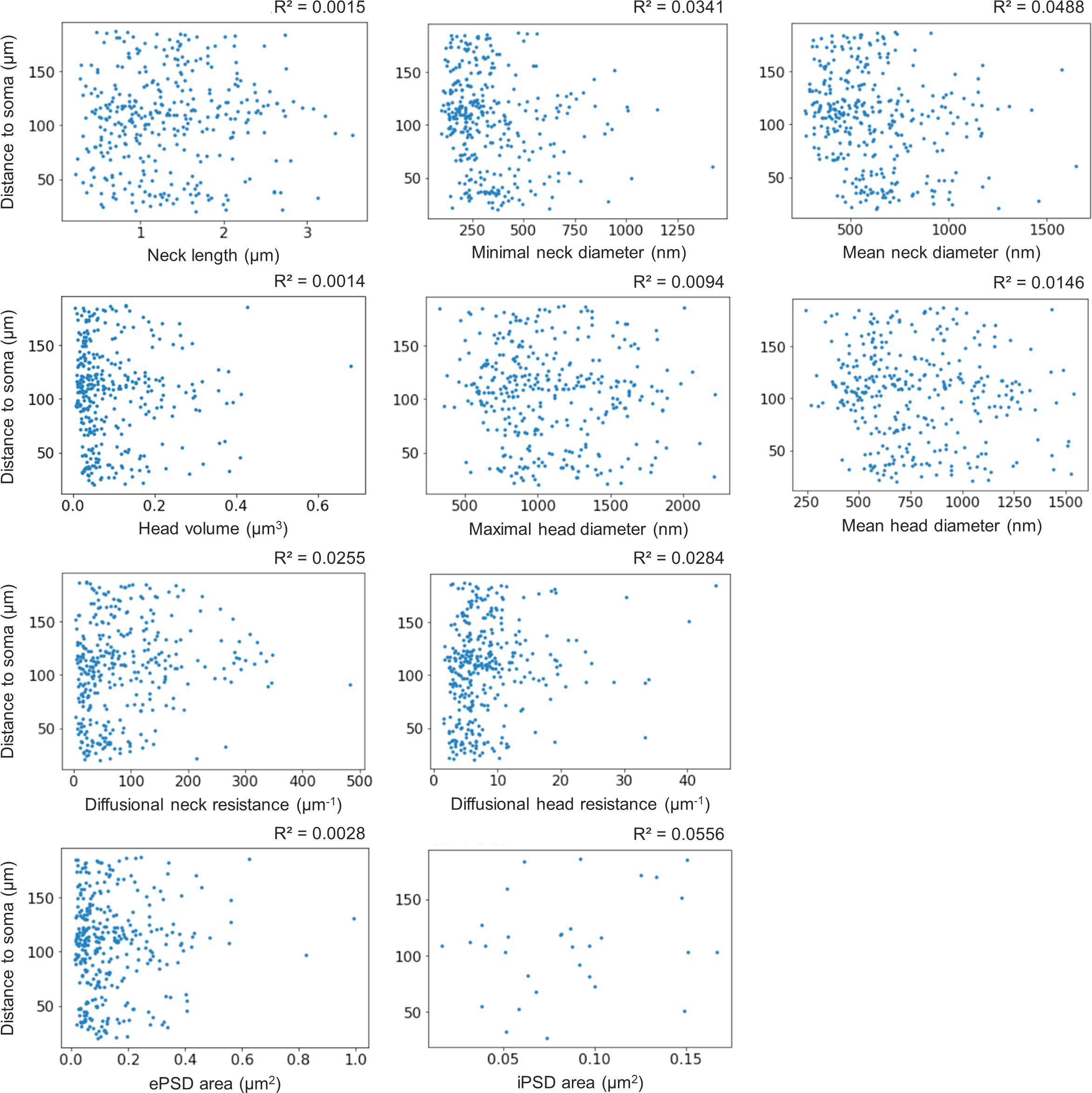

Supplement: S2 Fig — Distance between spine and soma as a function of all measured morphological parameters of the spines that were segmented. No parameter exhibited a linear correlation with the distance between spine and soma. The data underlying this figure can be found in https://www.opendata.bio.ens.psl.eu/3DCLEM-Spines/data/Data_related_to_FigS2.xlsx (login: guest; password: EnsData0811). ePSD, excitatory postsynaptic density; iPSD, inhibitory PSD. (TIF) [file pbio.3001375.s002.tif]

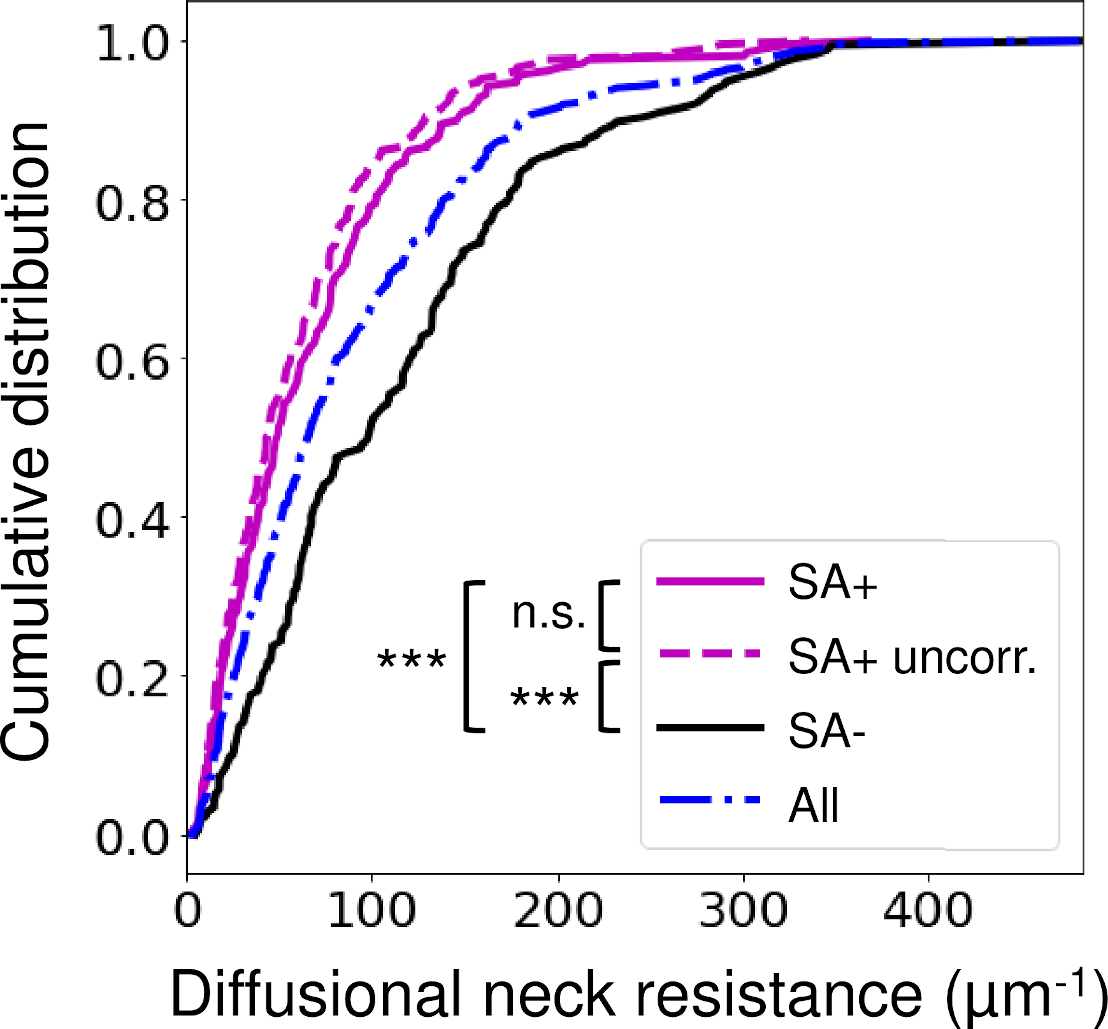

Supplement: S3 Fig — Distribution of the diffusional neck resistance (Wneck) calculated using neck morphology for spines devoid of apparatus (SA−) or containing an SA (SA+). “SA+ uncorr.”: Wneck without SA correction for SA+ spines (p = 0.1 compared to corrected Wneck). ***p < 0.001 calculated using Mann–Whitney test. The data underlying this figure can be found in https://www.opendata.bio.ens.psl.eu/3DCLEM-Spines/data/Data_related_to_FigS3.xlsx (login: guest; password: EnsData0811). n.s., not significant; SA, spine apparatus. (TIF) [file pbio.3001375.s003.tif]

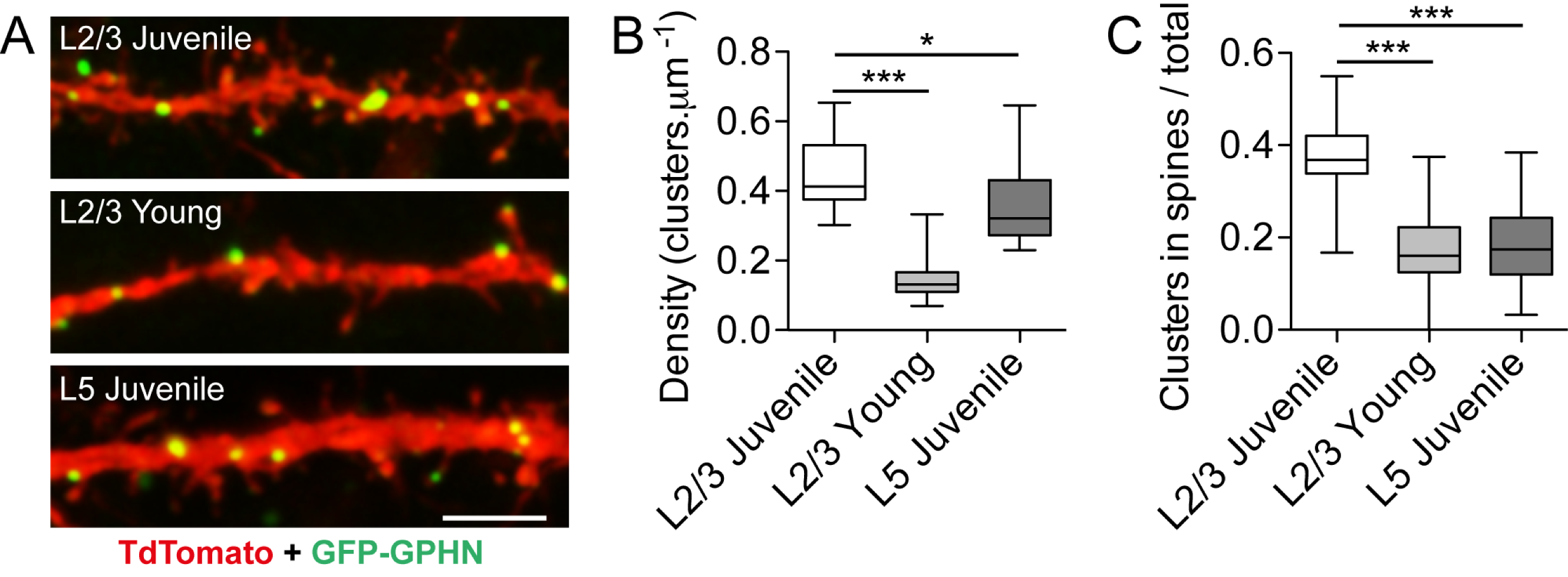

Supplement: S4 Fig — (A) Representative segments of dendrites of L2/3 and L5 PNs expressing GFP-GPHN (green) and TdTomato (red) in juvenile (postnatal days P21–P27) or young (P10) mice. Neuronal progenitors of L2/3 and L5 PNs were electroporated in utero at E15.5 and E12.5, respectively. Scale bar: 4 μm. (B, C) Quantification of gephyrin cluster density (B) and proportion of gephyrin clusters in spines (C). Data were acquired and analyzed as in our previous work [97]. Gephyrin cluster distribution was similar in adult (data in main text) and juvenile mice (density: 3.5 ± 1.1 clusters per 10 μm of dendrite in adults and 4.5 ± 1.0 clusters per 10 μm of dendrite in juveniles; proportion of gephyrin clusters in spines: 38% and 36% in adults and juveniles, respectively). However, both the density of gephyrin clusters and their proportion in spines was lower in younger (P10) mice, suggesting that DiSs represent mature spines (see also [103]). The proportion of gephyrin clusters in spines was also lower in L5 PNs than in L2/3 neurons. NL2/3 Juvenile = 18, NL2/3 Young = 28, NL5 Juvenile = 19. N represents the number of cells. Cells come from at least 3 animals per conditions. ***p < 0.001, *p < 0.05, ANOVA test followed by Tukey multiple comparisons test. The data underlying this figure can be found in https://www.opendata.bio.ens.psl.eu/3DCLEM-Spines/data/Data_related_to_FigS4.xlsx (login: guest; password: EnsData0811). DiSs, dually innervated spines; GFP-GPHN, GFP-tagged gephyrin; L2/3, layer 2/3; L5, layer 5; PN, pyramidal neuron. (TIF) [file pbio.3001375.s004.tif]

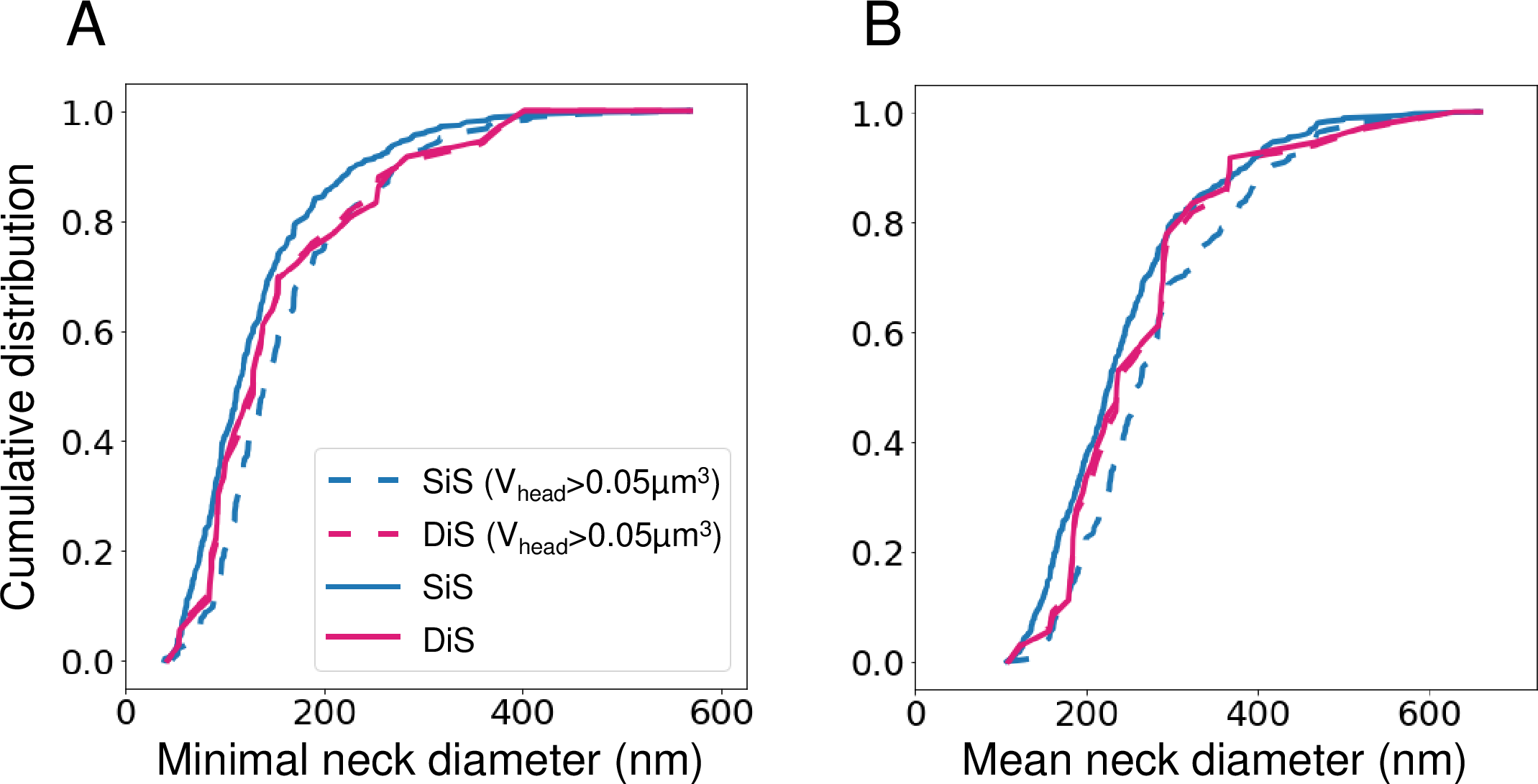

Supplement: S5 Fig — Quantification of minimal neck diameter (A) and mean neck diameter (B) for all spines (N = 349 SiSs and 37 DiSs; solid lines) and for spines with Vhead > 0.05 μm3 (N = 186 SiSs and 34 DiSs; dashed lines). p > 0.05 calculated using Mann–Whitney tests. The data underlying this figure can be found in https://www.opendata.bio.ens.psl.eu/3DCLEM-Spines/data/Data_related_to_FigS5.xlsx (login: guest; password: EnsData0811). DiSs, dually innervated spines; SiSs, singly innervated spines. (TIF) [file pbio.3001375.s005.tif]

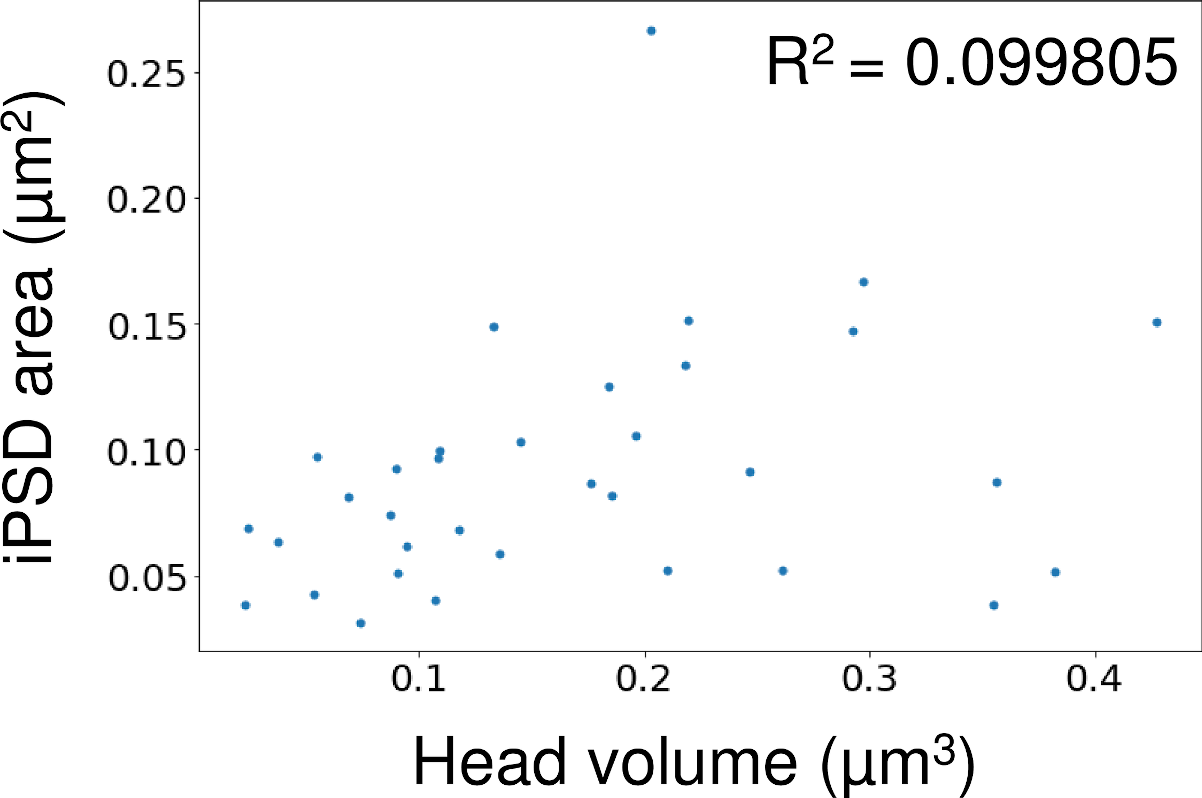

Supplement: S6 Fig — iPSD area as a function of spine head volume for N = 37 DiSs. Linear regression: R2 < 0.1. The data underlying this figure can be found in https://www.opendata.bio.ens.psl.eu/3DCLEM-Spines/data/Data_related_to_FigS6.xlsx (login: guest; password: EnsData0811). DiS, dually innervated spine; iPSD, inhibitory PSD. (TIF) [file pbio.3001375.s006.tif]

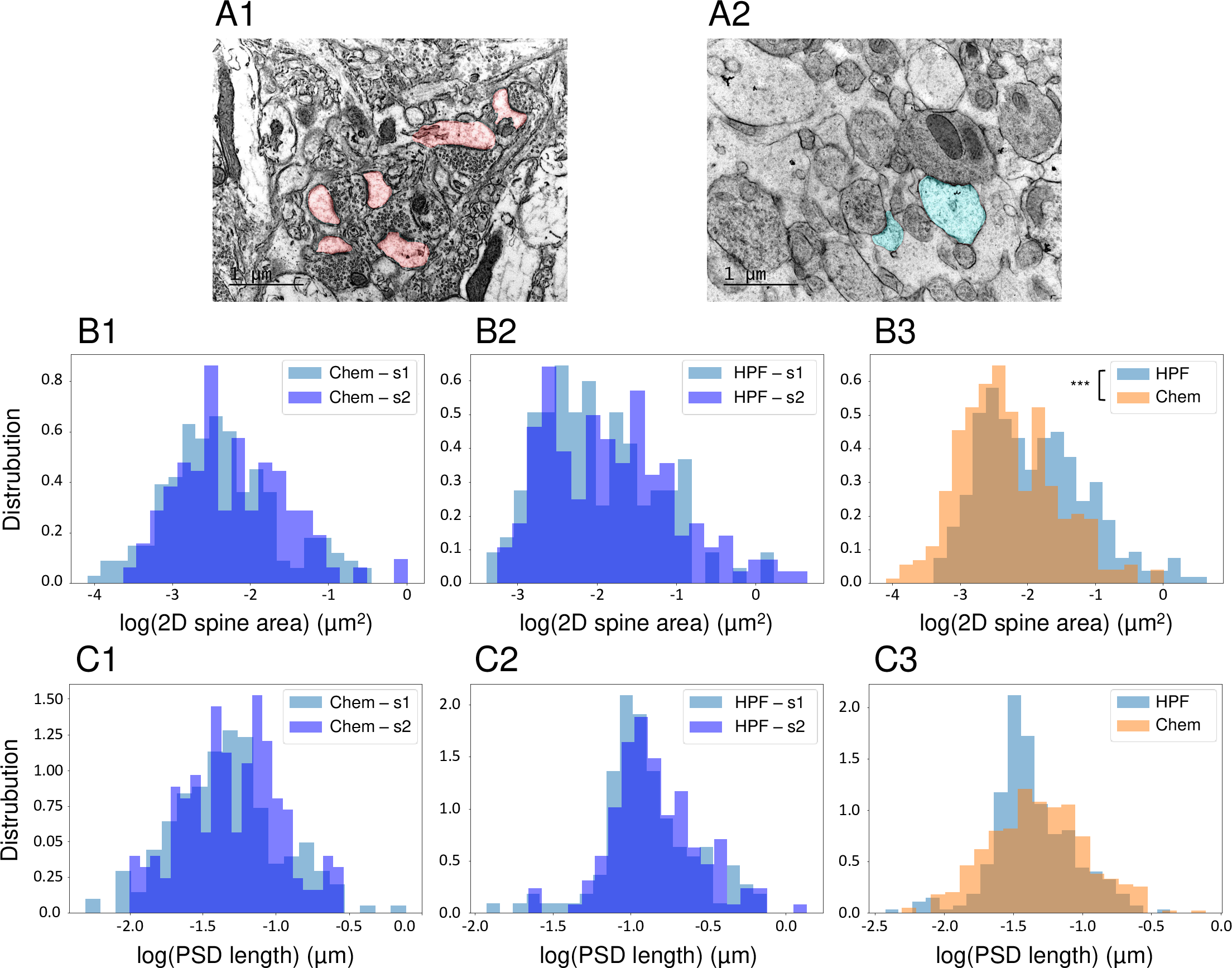

Supplement: S7 Fig — (A) TEM images of L2/3 SSC acute slices from the same mouse (postnatal day P21) upon either chemical fixation with aldehydes (A1) or physical fixation with HPF (A2). Spine head section areas (indicated in red in A1 and in light blue in A2) and lengths of PSDs were segmented for quantification. Scale bars: 1 μm. (B) Normalized histograms of spine head cross-section areas for chemically fixed (Chem) samples (N = 194 for mouse “s1” and 178 for mouse “s2”; p = 0.13) or HPF samples (N = 128 for s1 and 150 for s2; p = 0.052) in B1 and B2, respectively. Data from mice s1 and s2 displayed no statistical difference and were pooled together in B3 to compare area distribution between HPF (blue) and chemically fixed tissue (orange). Head cross-section areas were 34% ± 5% smaller in chemically fixed samples (orange) than in HPF samples (blue), implying approximately 45% head volume shrinkage (p < 10−8). (C) Normalized histograms of PSD section lengths for chemically fixed (p = 0.44) and HPF samples (p = 0.17) in C1 and C2, respectively. PSDs were not significantly deformed by chemical fixation (C3; p = 0.10). Only significant (p < 0.05) p-values are shown. ***p < 0.001 calculated using Mann–Whitney test. The data underlying this figure can be found in https://www.opendata.bio.ens.psl.eu/3DCLEM-Spines/data/Data_related_to_FigS7.xlsx (login: guest; password: EnsData0811). HPF, high-pressure freezing; L2/3, layer 2/3; PSD, postsynaptic density; SSC, somatosensory cortex; TEM, transmission electron microscopy. (TIF) [file pbio.3001375.s007.tif]

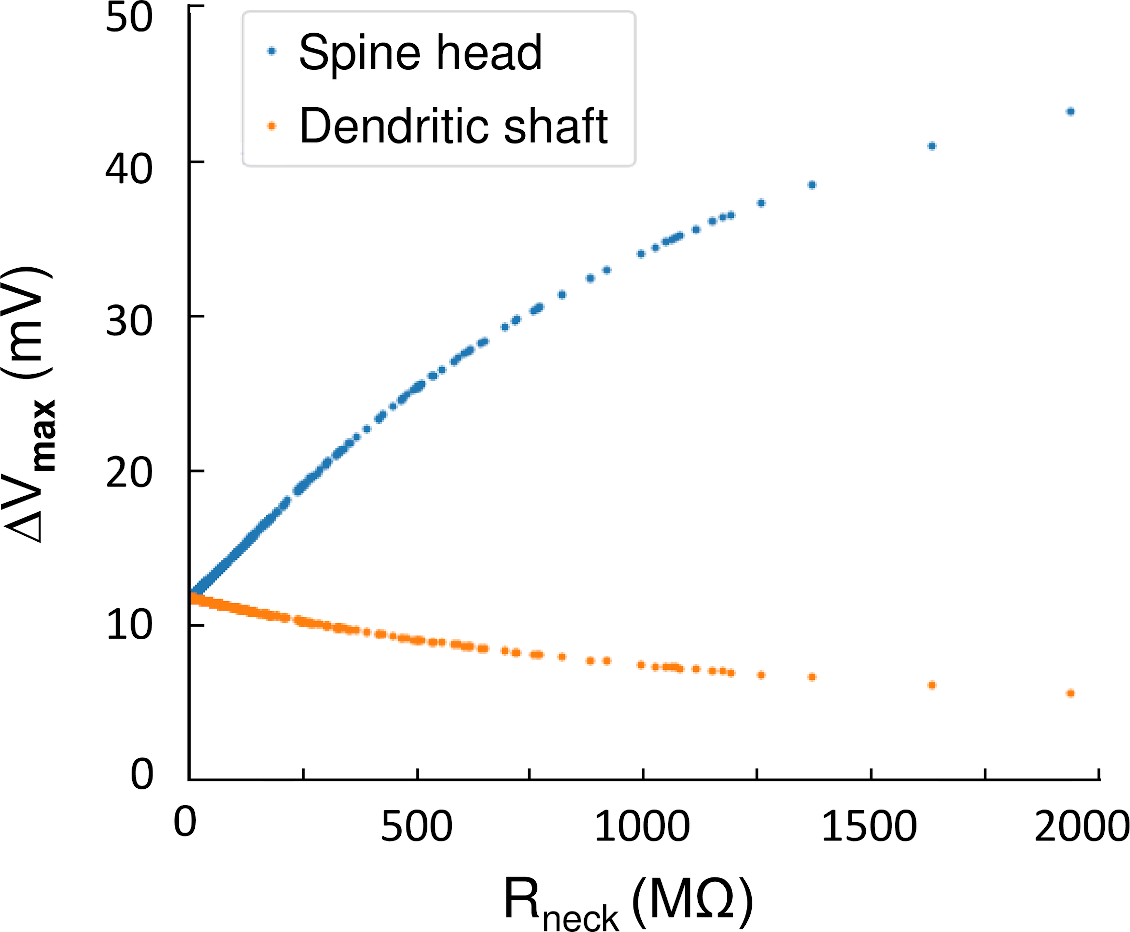

Supplement: S8 Fig — Plot of the EPSP amplitude ΔVmax elicited in a dendritic spine while varying its neck resistance (Rneck) and keeping all other parameters constant. Increasing Rneck causes ΔVmax to increase in the spine head (blue) and to decrease in the dendritic shaft (orange). The data underlying this figure can be found in https://www.opendata.bio.ens.psl.eu/3DCLEM-Spines/data/Data_related_to_FigS8.xlsx (login: guest; password: EnsData0811). EPSP, excitatory postsynaptic potential. (TIF) [file pbio.3001375.s008.tif]

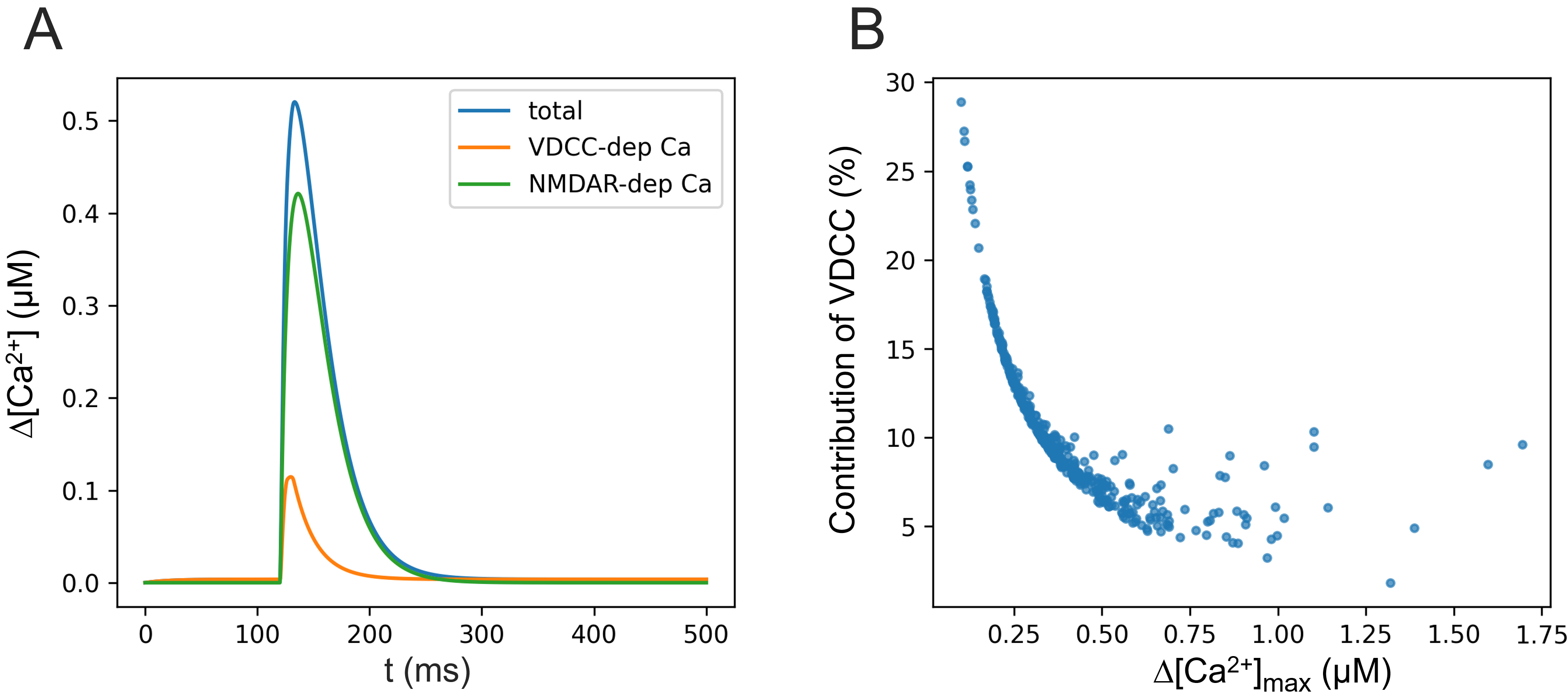

Supplement: S9 Fig — (A) Typical time course of a calcium transient elicited in spine head. The calcium signal was induced by an EPSP at t = 120 ms. The contributions of VDCCs and NMDARs to the total elevation of calcium concentration (blue curve) are plotted in orange and green, respectively. (B) Contribution of VDCCs to the total elevation of calcium concentration as a function of the estimated amplitude of the calcium transient in all spines (N = 390). The data underlying this figure can be found in https://www.opendata.bio.ens.psl.eu/3DCLEM-Spines/data/Data_related_to_FigS9.xlsx (login: guest; password: EnsData0811). EPSP, excitatory postsynaptic potential; NMDAR, NMDA receptor; VDCC, voltage-dependent calcium channel. (TIF) [file pbio.3001375.s009.tif]

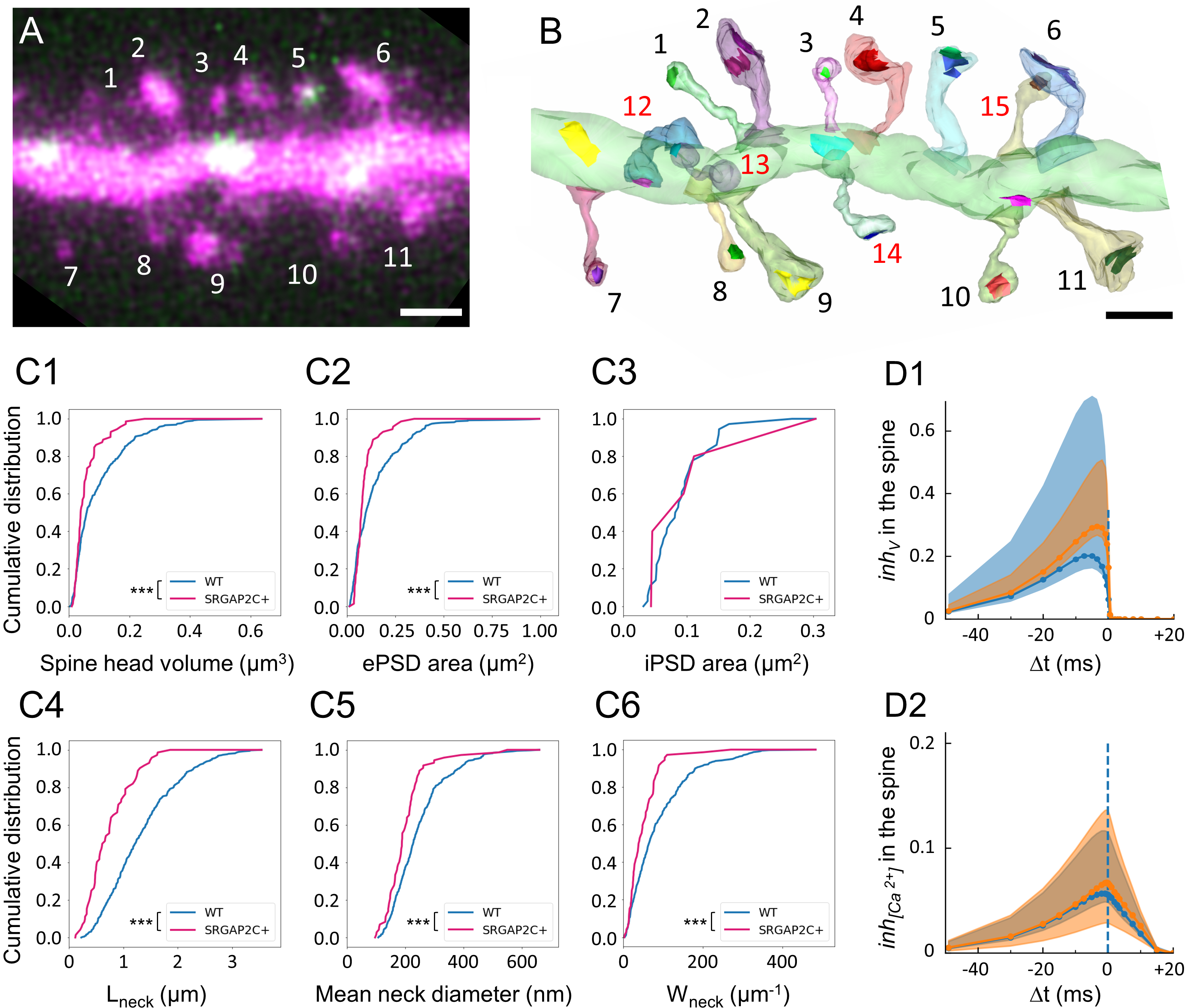

Supplement: S10 Fig — (A) Z-projection of the confocal stack showing a segment of basal dendrite of an L2/3 PN expressing cytosolic TdTomato (in magenta), GFP-GPHN (in green), and SRGAP2C in the adult mouse SSC. Neurons were electroporated in utero at E15.5. Numbers identify individual spines. Scale bar: 1 μm. (B) 3D-EM reconstruction. Individual dendritic spines are manually segmented and randomly colored. Spines that were detected in CLEM but not in LM alone are labelled in red. Scale bar: 1 μm. (C) Quantification of spine head volume (C1), ePSD area (C2), DiS–iPSD area (C3), neck length (Lneck) (C4), mean neck diameter (C5), and the diffusional neck resistance (Wneck) (C6) for WT spines (blue) and spines expressing SRGAP2C (red). N = 73 spines expressing SRGAP2C and 390 WT spines in panels C1–C2 and C4–C6. N = 6 DiSs expressing SRGAP2C and 37 WT DiSs in panel C3. ***p < 10−3 for all quantities except for iPSD area, Mann–Whitney test. Comparison with our previous work on oblique apical dendrites [97,127] suggests that SRGAP2C expression has dendrite-type specific consequences on spine density and morphology. (D) Similar panels as in Fig 7 but for spines along the basal dendrites of neurons expressing SRGAP2C. (D1) Voltage inhibition in the spine head, inhV, induced by dendritic (blue) or spinous (orange) IPSPs as a function of Δt. (D2) Inhibition of the calcium influx in the spine head, inh[Ca2+], induced by dendritic (blue) or spinous (orange) IPSPs as a function of Δt. The inhibition of calcium signals was approximately 40% lower in neurons expressing SRGAP2C than in control neurons (Fig 7). The data underlying this figure can be found in https://www.opendata.bio.ens.psl.eu/3DCLEM-Spines/data/Data_related_to_FigS10.xlsx (login: guest; password: EnsData0811). CLEM, correlative light–electron microscopy; DiS, dually innervated spine; ePSD, excitatory postsynaptic density; GFP-GPHN, GFP-tagged gephyrin; iPSD, inhibitory PSD; IPSP, inhibitory postsynaptic potential; LM, light microscopy; [file pbio.3001375.s010.tif]
